# FLAME: Training and Validating a Newly Conceived Model Incorporating Alpha-Glutathione-S-Transferase Serum Levels for Predicting Advanced Hepatic Fibrosis and Acute Cardiovascular Events in Metabolic Dysfunction-Associated Steatotic Liver Disease (MASLD)

**DOI:** 10.3390/ijms26020761

**Published:** 2025-01-17

**Authors:** Marcello Dallio, Mario Romeo, Fiammetta Di Nardo, Paolo Vaia, Carmine Napolitano, Lorenzo Ventriglia, Annachiara Coppola, Alessia Silvestrin, Simone Olivieri, Alessandro Federico

**Affiliations:** Hepatogastroenterology Division, Department of Precision Medicine, University of Campania Luigi Vanvitelli, Piazza Miraglia 2, 80138 Naples, Italy; marcello.dallio@unicampania.it (M.D.); fiammetta.dinardo@studenti.unicampania.it (F.D.N.); paolo.vaia@studenti.unicampania.it (P.V.); carmine.napolitano1@studenti.unicampania.it (C.N.); lorenzo.ventriglia@unicampania.it (L.V.); annachiara.coppola@unicampania.it (A.C.); alessia.silvestrin@unicampania.it (A.S.); simone.olivieri@policliniconapoli.it (S.O.); alessandro.federico@unicampania.it (A.F.)

**Keywords:** liver cirrhosis, biomarkers, fatty liver

## Abstract

Alpha-Glutathione-S-transferase (alphaGST) is a liver enzyme whose serum levels increase with the worsening of fibrosis in alcoholic and viral chronic hepatitis. Its usefulness in Metabolic Dysfunction-Associated Steatotic Liver Disease (MASLD) remains unexplored. From January 2016 to December 2017, 200 patients with MASLD and 30 controls were enrolled. AlphaGST serum levels were measured. Variables related to advanced fibrosis (AF) were selected via Principal Component Analysis (PCA), and the best cut-off (BCO) was estimated using ROC analysis. Liver stiffness measurement (LSM), NAFLD fibrosis (NFS), Fibrosis-4 (FIB-4), and BMI-AST/ALT Ratio-Diabetes (BARD) scores were determined. The first acute cardiovascular events (ACE) in ACE-naïve subjects were recorded over five years. A validation cohort of 60 MASLD patients was enrolled from January 2018 to May 2019 and followed for five years. AlphaGST levels increased with fibrosis stage (*p* < 0.0001) in both cohorts, showing high accuracy in predicting AF (TrC: AUC 0.89, VlC: AUC 0.89). PCA-selected variables were HbA1c, HDL, and alphaGST, forming the “FLAME” model. FLAME showed superior predictive performance for AF and ACEs compared to other models and scores. FLAME represents a novel tool that accurately predicts AF and ACEs in MASLD.

## 1. Introduction

Alpha-Glutathione-S-transferases represent the most relevant component of the complex “Glutathione-S-transferase” isoenzyme family, including even Pi, Sigma, Theta, Omega, and Zeta, ubiquitously expressed by various human cell types [1]. In particular, alphaGSTA1 (alphaGST) constitutes up to 3% of hepatocyte cytosolic protein content and is actively implicated in diverse detoxifications and anti-oxidative stress pathways [1]. Considering its homogeneous distribution across the lobule structure and differently from other typically used cell injury indicators predominantly localized in the periportal hepatocytes, alphaGST has been identified as an early and sensitive damage biomarker [1,2,3]. For this purpose, its serum levels have already been reported to progressively increase in viral and alcoholic chronic hepatitis contexts, according to the worsening of liver fibrosis [4]. However, its clinical usefulness in patients with Non-alcoholic Fatty Liver Disease (NAFLD), more recently redesigned as Metabolic dysfunction-associated Steatotic Liver Disease (MASLD), has never been explored [5].

MASLD, encompassing a spectrum of disease manifestations ranging from simple steatosis (SS) to steatohepatitis (MASH), advanced fibrosis (AF), and liver cirrhosis [6], currently represents the most common cause of chronic liver damage in Western countries, due to the spread of unhealthy lifestyle habits [7]. The pathogenetic triad for its development and worsening, represented by inflammation, oxidative stress, and insulin resistance (IR), involves several biological and tangled network systems, implicating the strict and not fully elucidated connections between environment, genetics, gut microbiota, and immune reactivity [8,9].

Relevantly, AF-affected patients (F3 and F4 according to Metavir score) show a high risk of hepatocellular carcinoma (HCC) and development of extrahepatic complications, as well as increased mortality due to both liver and metabolic syndrome (MS)-related cardiovascular events [10]. For this reason, early fibrosis identification and, consequently, patient risk stratification, represent the basement to design the more appropriate tailored approach for the optimal follow-up and multidisciplinary clinical management strategy.

Several composite scores (e.g., NAFLD Fibrosis score, NFS; Fibrosis-4, Fib-4; BMI-AST/ALT Ratio-Diabetes score, BARD) [11] have already been proposed to non-invasively identify AF, minimizing the need for liver biopsies in clinical practice [11]. However, despite the overall good accuracy in predicting the upper and lower ranges of liver fibrosis degree, all these scores are affected by a huge gray zone of dropped accuracy for the intermediate stage’s identification [11]. Moreover, no routine biomarkers or non-invasive tools differentiating SS from MASH are currently available.

In this study, we primarily aimed to evaluate the correlation between alphaGST levels in MASLD-affected patients and the disease stage by assessing its role and accuracy as a potential biomarker for the identification of AF. Furthermore, by including the biochemical variables significantly and highly associated with AF, we aimed to design a new tool incorporating alphaGST. Finally, in light of the largely reported close physiopathological link connecting AF with the systemic associated dysmetabolic abnormalities and high cardiovascular risk in this setting of patients [12,13], we aimed to evaluate the accuracy of the newly conceived “Free plasma glucose/insulin resistance-related abnormalities—Lipid-Associated Metabolic alterations—Excretion of liver-injuring toxic metabolites/anti-oxidative stress mechanisms—impairment (FLAME)” index in the prediction of AF in MASLD patients and the 5-year first atherosclerosis-related acute cardiovascular event (ACE) occurrence in ACE-naïve MASLD individuals.

## 2. Results

### 2.1. Training Cohort

Two hundred MASLD patients were enrolled in the Training Cohort (TrC). Of these, 12 were lost (dead of extrahepatic and extra-cardiovascular events), whereas 188 patients completed the follow-up period. 30 healthy controls were also recruited.

The baseline demographic data, anthropometric indexes, biochemical parameters, and non-invasive tools for liver fibrosis assessment (LSM, NFS, FIB-4, and BARD) of the study population (TrC and healthy controls) are reported in Table 1.

#### 2.1.1. AlphaGST and Baseline Liver Fibrosis

The percutaneous liver biopsy (pLB) on TrC-MASLD patients revealed an initial fibrosis stage (F0–F2, Ishak score: 0–3) for 90/200 (45%) and AF (F3–F4, Ishak score > 3) for 110/200 (55%) patients, of which 60 (30%) showed F3 (Ishak score: 4–5) and 50 (25%) F4 (Ishak score: 6) stage.

**Figure 1:**
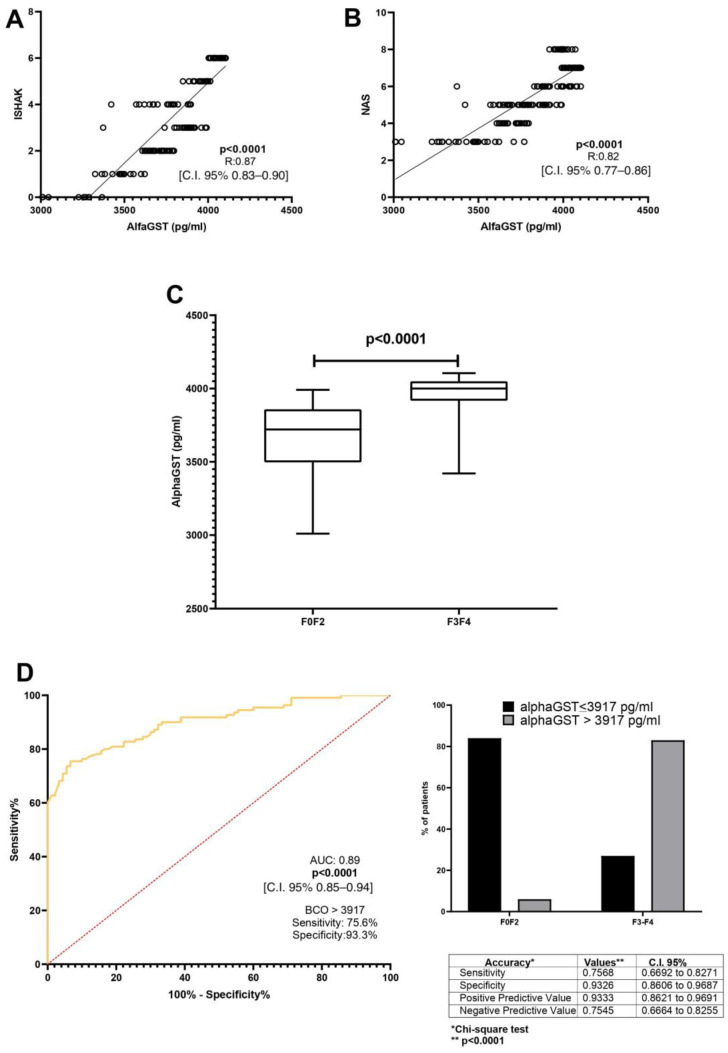
AlphaGST levels and histologically-assessed liver fibrosis. (**A**): Relationship between alphaGST levels and Ishak staging system score (Linear regression analysis). (**B**): Relationship between alphaGST levels and NAFLD Activity score (NAS) (Linear regression analysis). (**C**): AlphaGST levels in initial vs. advanced fibrosis stage (F3–F4) (Mann–Whitney test). (**D**): Diagnostic accuracy of AlphaGST levels in predicting histologically assessed Advanced Fibrosis (F3–F4). Receiver operator characteristic (ROC) curve analysis using the Youden index to determine the AlphaGST best cut-off value in the identification of F3–F4 patients; Chi-square test. BCO: best cut-off; C.I.: Confidence Interval.

A direct correlation between alphaGST levels (pg/mL) and the Ishak total score [*p* < 0.0001, R: 0.87, C.I. 95% 0.83–0.90] was demonstrated. Moreover, alphaGST levels (pg/mL) were directly related to the NAFLD activity score (NAS) [*p* < 0.0001, R: 0.82, C.I. 95% 0.77–0.86] (Figure 1A,B). The mean level of the AlphaGST resulted in more elevated in AF-affected patients (F3–F4), compared to F0–F2 patients (*p* < 0.0001) (Figure 1C). ROC analysis with the Youden index calculation for the identification of best cut-off (BCO) values showed the AlphaGST value of 3917 pg/mL [AUC: 0.89, *p* < 0.0001, C.I. 95% 0.85–0.94, sensitivity: 75.68%, specificity: 93.26%, NPV:75.45%, PPV:93.33%] as the best cut-offs to identify liver biopsy assessed AF (Figure 1D).

#### 2.1.2. FLAME Index: Variables Selection, Composition, and Determination

Principal Component Analysis (PCA) identified the following biochemical variables for each predefined dimension (PC) when the histological diagnosis of AF was considered: glycosylated hemoglobin (HbA1c) (%), high-density lipoprotein (HDL) (mg/dL), and alphaGST (pg/mL) (Supplementary Figure S1A–C). ROC determined the relative BCO for each value: HbA1c > 5.5% (AUC: 0.56), HDL < 43.5 mg/dL (AUC: 0.58), and alphaGST levels > 3917 pg/mL (AUC: 0.89) (Supplementary Figure S1D). The relative “weight” of the accuracy (AUC) was subsequently considered: for variables whose AUC < 0.80, the overcoming of the ROC-identified BCO scored “2 points” rather than “1 point”; contrariwise, for variables whose AUC > 0.80, the overcoming of the ROC-identified BCO scored “4 points” rather than “2 points”. Based on this, the FLAME index was calculated as the sum of (A) + (B) + (C) as reported in Table 2.

As appreciable, the FLAME index total score ranges from a minimum of 4 to a maximum of 8 points. Supplementary Figure S1E reports the scientific rationale linking reciprocally the selected variables with AF, supporting the PCA- and ROC-obtained results.

#### 2.1.3. FLAME and Baseline Liver Fibrosis

**Figure 2:**
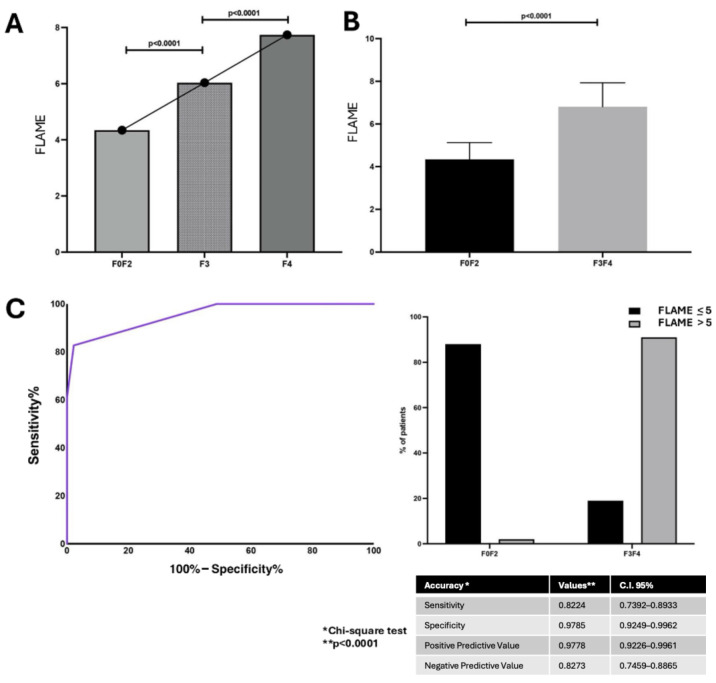
The FLAME index and histologically assessed liver fibrosis relationship. (**A**): FLAME index total scores in different fibrosis stages. (ANOVA). (**B**): Box-plot comparison of FLAME index total scores main levels between mild (F0–2) and advanced fibrosis stages (F3–4) (Mann–Whitney test). (**C**): Diagnostic accuracy of FLAME index in predicting histological Advanced Fibrosis (F3–F4). Receiver operator characteristic (ROC) curve analysis using the Youden index to determine the FLAME index best cut-off value in the identification of F3–F4 patients; Chi-square test. BCO: best cut-off; C.I.: Confidence Interval.

The linear regression analysis revealed a positive correlation between the FLAME index total score and Ishak score [*p* < 0.0001 R: 0.830; C.I. 95% 0.78–0.87]. The FLAME index total score ranged from 4 to 8 points and, consistently with alphaGST levels’ trend, was shown to progressively increase in AF patients (F3–F4) compared to F0–F2 ones (*p* < 0.0001) (Figure 2A,B).

ROC analysis with the Youden index calculation for the identification of best cut-off values revealed the FLAME index threshold of 5 as the BCO [AUC: 0.95, *p* < 0.0001, C.I. 95% 0.92–0.97, sensitivity: 82.7%, specificity: 97.8%, NPV: 82.2%, PPV: 97.8%] for the identification of histologically assessed AF patients (F3–F4) (Figure 2C).

Furthermore, the linear regression analysis highlighted the significant correlation of the FLAME index with NFS [*p* < 0.0001, R: 0.640, C.I. 95% 0.54–0.71], FIB-4 [*p* < 0.0001, R: 0.695, C.I. 95% 0.61–0.76], and BARD score [*p* < 0.0001, R: 0.759, C.I. 95% 0.68–0.81].

**Figure 3:**
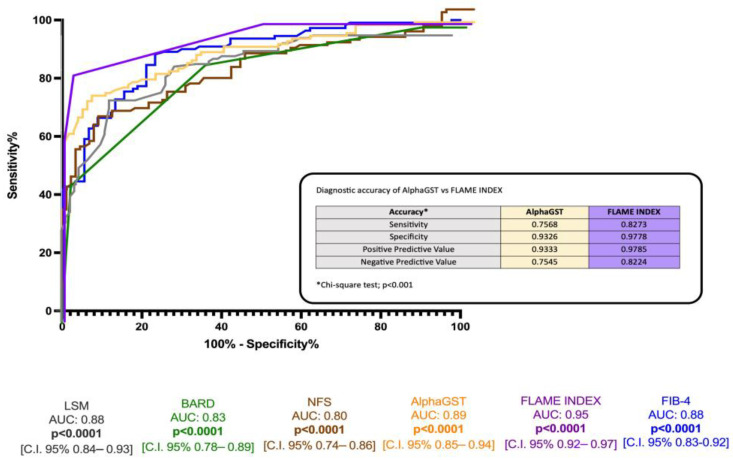
Diagnostic accuracy comparison of the alphaGST levels, FLAME index, and other non-invasive composite tools in the prediction of liver biopsy-assessed advanced fibrosis. Receiver operator characteristic (ROC) curve of alphaGST (orange), FLAME index (purple), NFS (brown), FIB-4 (blue), BARD (green), LSM (gray). In the box, head-to-head comparison of alphaGST levels vs. FLAME Index diagnostic accuracy. NFS: NAFLD fibrosis score; FIB-4: Fibrosis-4; BARD: BMI-AST/ALT Ratio-Diabetes. LSM: Liver stiffness measurement.

ROC analysis revealed the better accuracy of the FLAME index (BCO > 5) compared to alphaGST alone [AUC: 0.89], NFS [AUC: 0.80], FIB-4 [AUC: 0.88], BARD score [AUC 0.83], and LSM [0.88] in the identification of histologically assessed AF (Figure 3).

#### 2.1.4. Acute Cardiovascular Events

Of 200 MASLD-enrolled individuals, 12 were lost (5 F0–F2 and 7 AF individuals dead of extrahepatic and extra-cardiovascular events) and were thus excluded from the analysis, as well as twenty-eight not ACE-naïve patients. The frequency distribution of ACEs over the 5-year follow-up according to the baseline fibrosis stage is reported in Supplementary Table S1A.

**Figure 4:**
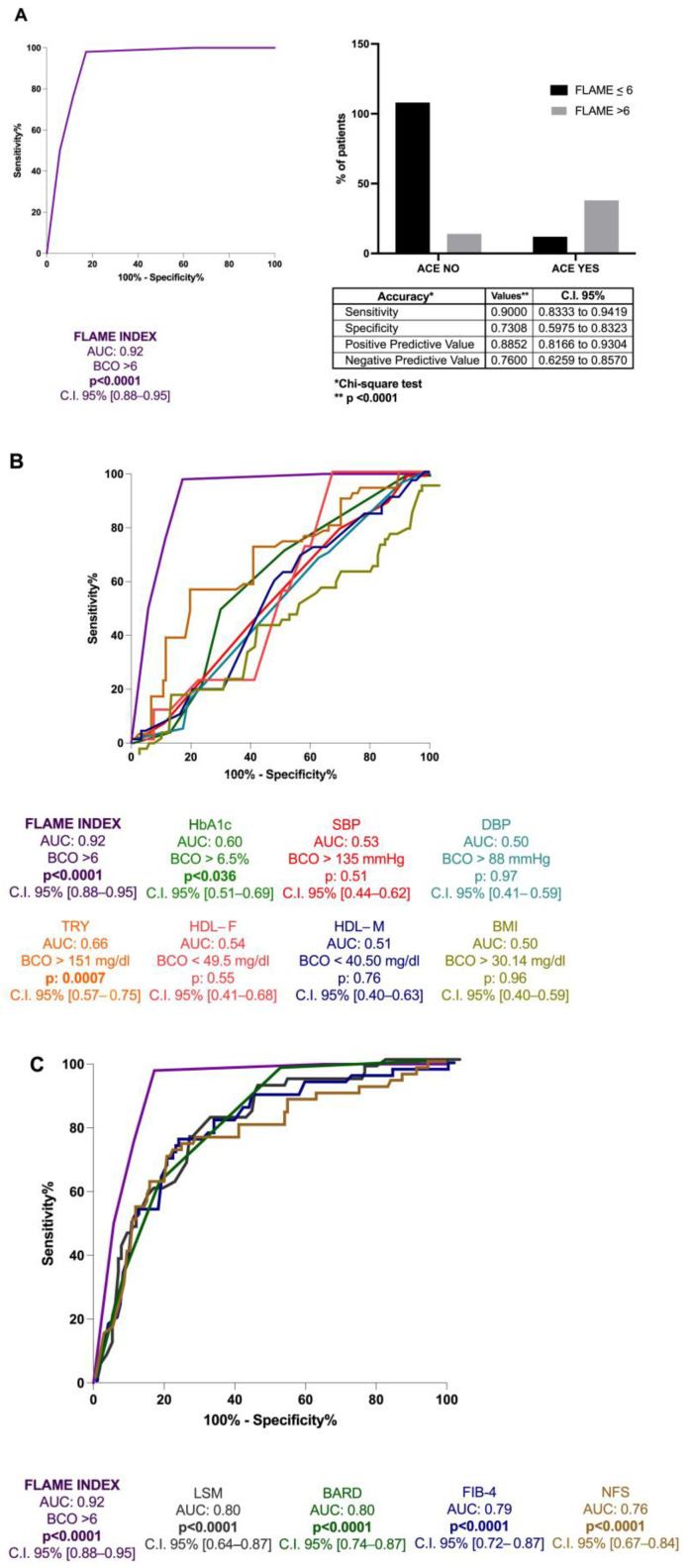
The FLAME index and ACEs occurrence in MASLD patients’ relationship. (**A**): Diagnostic accuracy of FLAME index in predicting ACEs occurrence at 5 years in MASLD patients. Receiver operator characteristic (ROC) curve analysis using the Youden index to determine the FLAME index best cut-off value in the prediction of the outcome; Chi-square test. *BCO: best cut-off; C.I.: Confidence Interval.* (**B**): Diagnostic accuracy comparison of the FLAME index and other hematochemical and anthropometrical parameters analytically measuring the “modifiable” cardiovascular risk factors. Receiver operator characteristic (ROC) of FLAME index (purple), HbA1c (green), SBP (red), DBP (water-green), triglycerides (orange), HDL-female (pink), HDL-male (blue), BMI (yellow). HbA1c: glycosylated hemoglobin; SBP: Systolic blood pressure; DBP: Diastolic blood pressure; TRY: triglycerides; HDL-F: High-density lipoprotein female; HDL-M: High-density lipoprotein male; BMI: Body Mass Index. (**C**): Diagnostic accuracy comparison of the FLAME index and other non-invasive composite tools in the prediction of ACEs occurrence at 5 years. Receiver operator characteristic (ROC) of FLAME index (purple), NFS (brown), FIB-4 (blue), BARD (green), LSM (gray). NFS: NAFLD fibrosis score; FIB-4: Fibrosis-4; BARD: BMI-AST/ALT Ratio-Diabetes. LSM: Liver stiffness measurement.

ROC analysis with the Youden index calculation for the identification of BCO values revealed the FLAME index threshold of 6 [AUC: 0.92, *p* < 0.0001, C.I. 95% 0.88–0.95, sensitivity: 90%, specificity: 73%, NPV: 76%, PPV: 88.5%] for the 5 years prediction of the first ACE occurrence (Figure 4A). Moreover, its better predictive performance in comparison to hematochemical [HDL (AUC:0.54 for male and 0.51 for female), triglycerides (TG) (AUC: 0.66), and HbA1c (AUC: 0.60)], anthropometrical parameters [body mass index (BMI) (AUC: 0.50), systolic blood pressure (SBP) (AUC: 0.53), and diastolic blood pressure (DBP) (AUC: 0.50)] and the other non-invasive composite tools for fibrosis assessment [NFS (AUC: 0.76), FIB-4 (AUC: 0.79), BARD score (AUC: 0.80), and LSM(AUC: 0.80)] (all *p* < 0.0001) (Figure 4B,C) in the prediction of the first ACE was highlighted.

The multinomial logistic regression analysis adjusted for the variables [sex, age, BMI, diabetes, arterial hypertension, Mediterranean diet compliance, physical exercise, smoking, drugs administration (including anticoagulant/antiaggregant, statins, and GLP1-Receptor Agonist- GLP1-RA), and alcohol intake] revealed the FLAME index as the only variable significantly associated with the 5-year occurrence of the first ACE for MASLD patients (Supplementary Table S2).

**Figure 5:**
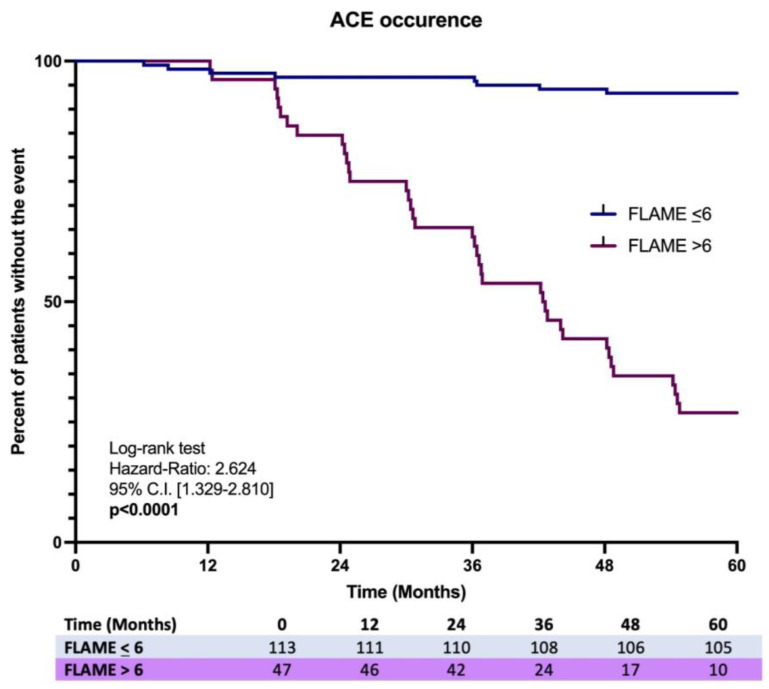
Kaplan-Meir analysis on ACEs occurrence in MASLD patients with the comparison of the incidence proportion rate between baseline FLAME ≤ 6 vs. baseline FLAME > 6 individuals.

Finally, the Kaplan–Meier analysis on the first ACE occurrence over 5 years showed a significant difference in the incidence ratio rate (IRR) (7.1% vs. 78.2%), revealing a higher risk in MASLD patients having a baseline FLAME index > 6 [Log-rank test, HR: 2.624, C.I. 95% 1.329–2.810, *p* < 0.0001] (Figure 5).

#### 2.1.5. Liver-Related Events

Respectively, the onset of CSPH was reported in 14 of 85 (16.5%) baseline F0–F2 and 61 of 103 (59.2%) baseline F3–F4 individuals completing the follow-up.

Regarding the first LRE’s occurrence prediction during the 5-year follow-up, ROC analysis revealed a similar performance of the FLAME index [AUC: 0.721] compared to NFS [AUC: 0.719], FIB-4 [AUC: 0.720], BARD [AUC: 0.713], and LSM [AUC: 0.721]. Detailed data concerning LRE type and relative frequency distributions according to the baseline fibrosis stage are reported in Supplementary Table S3A.

### 2.2. Validation Cohort

Sixty MASLD patients were enrolled in the Validation Cohort (VlC), and all the individuals completed the 5-year follow-up period. The baseline demographic data, anthropometric indexes, biochemical parameters, and non-invasive tools for liver fibrosis assessment (LSM, NFS, FIB-4, and BARD) of the VlC are reported in Table 1. 

The pLB on VlC-MASLD patients revealed an initial fibrosis stage (F0–F2, Ishak score: 0–3) for 27/60 (45%) and advanced fibrosis (F3–F4, Ishak score > 3) for 33/60 (55%) patients, of which 15 (25%) showing F3 (Ishak score: 4–5) and 18 (30%) F4 (Ishak score: 6). AlphaGST levels (pg/mL) were higher in AF-affected patients (F3–F4), compared to F0–F2 patients (*p* < 0.0001) showing a direct positive correlation with both NAS and the Ishak total score [NAS: *p* < 0.0001, R:0.76, C.I. 95%: 0.73–0.85; Ishak: *p* < 0.0001, R: 0.76, C.I. 95%: 0.71–0.88] and an elevated accuracy in the prediction of AF [AUC:0.89, C.I. 95%: 0.80–0.97, *p* < 0.0001] (Supplementary Figure S2).

FLAME index total score progressively increased in AF patients (F3–F4) patients compared to F0–F2 ones (*p* < 0.0001), presenting a direct positive correlation with both NAS and the Ishak total score [NAS: *p* < 0.0001, R: 0.74, C.I. 95%: 0.70–0.83; Ishak: *p* < 0.0001, R: 0.88, C.I. 95%: 0.81–0.93] (Supplementary Figure S3), as well as a higher accuracy in the prediction of AF [AUC: 0.94; C.I. 0.89–0.98, *p* < 0.0001; BCO >5] in comparison to alphaGST alone [AUC: 0.89, *p* < 0.0001] and the other non-invasive tools [NFS (AUC: 0.79), FIB-4 (AUC: 0.85), BARD (AUC: 0.81), and LSM (AUC: 0.83), all *p* < 0.0001], confirming the results observed in the TrC.

Concerning ACEs, of 60 VlC-MASLD patients, 6 individuals were not naïve, presenting at least one previous event, and were thus excluded from the analysis (Supplementary Table S1B). The FLAME index presented a higher accuracy [AUC: 0.91, *p* < 0.0001, C.I. 95% 0.84–0.92; BCO > 6] compared with hematochemical [HDL (AUC: 0.53 for male and 0.51 for female), TG (AUC: 0.62), and HbA1c (AUC: 0.57)], anthropometrical parameters [BMI (AUC: 0.68), SBP (AUC: 0.54), and DBP (AUC: 0.51)], and the other non-invasive composite tools for liver fibrosis assessment [NFS (AUC: 0.86), FIB-4 (AUC: 0.89), BARD score (AUC: 0.82), and LSM (AUC: 0.80)] in the prediction of the 5 year first ACE.

Finally, the onset of CSPH was observed in 5 of 27 (18.5%) baseline F0–F2 and 18 of 33 (54.5%) baseline F3–F4 individuals. Regarding the first LRE occurrence (Supplementary Table S3B), a similar performance of the FLAME index [AUC: 0.78] compared to NFS [AUC: 0.75], FIB-4 [AUC: 0.74], BARD [AUC: 0.72], and LSM [AUC: 0.73] in predicting this outcome was reported.

## 3. Discussion

In light of the current epidemiologic revolution of hepatology due to the spread of metabolic disorders, the scientific community all over the world has been completely involved in finding new therapeutic opportunities for MASLD [14,15,16]. Nevertheless, the progress of diagnostic accuracy supporting the correct patient risk stratification and tailored management program represents one of the biggest challenges of modern hepatology [17,18].

Up to now, the most important prognostic factor in this context has been represented by liver fibrosis deposition, affecting both hepatic and extrahepatic complications onset, as well as influencing the therapeutic response [19,20,21].

During the last decade, diverse serologically and radiologically evaluable surrogates for the non-invasive prediction of liver fibrosis were identified and their routine application become an ordinary habit [22,23]. In line with the latter concept, their accessibility, repeatability, and lower cost compared to pLB were identified as crucial characteristics for their use at a population level [23].

The alphaGST represents an isoform of GST protein, largely distributed across the liver lobule, where it is involved in various detoxification processes [1,2]. Over the years, its clinical significance was mainly referred to the use as an early marker of alcoholic or viral-mediated liver damage, comparing its accuracy in this sense with other largely used makers of the long-gone past [3,4]. However, its role in this purpose in MASLD-related liver damage was not largely explored and, in particular, no strong scientific evidence on its correlation with histological changes and disease progression has been published before. Here, we demonstrated the direct and linear correlation between alphaGST blood levels and the histologically assessed fibrosis progression, confirming these results in a VlC.

In particular, by using the ROC analysis, we pointed out the limit of 3917 pg/mL as a clinically significant diagnostic threshold to differentiate mild fibrosis (F0–2) from advanced fibrosis (≥F3) with a good diagnostic accuracy confirmed by specificity, sensitivity, PPV, and NPV in TrC.

In this cohort, the multivariate analysis (PCA) was adopted to identify and select the biochemical variables for each predefined dimension (PC) [“Free plasma glucose/insulin resistance-related abnormalities—Lipid-Associated Metabolic alterations—Excretion of liver-injuring toxic metabolites/anti-oxidative stress mechanisms—impairment”] when the histological diagnosis of AF was considered. By adopting the relative ROC-determined BCOs, the “FLAME” index was conceived, and the relative calculation was configured (Supplementary Figure S1). Interestingly, the obtained BCOs for the considered variables consistently reflected those currently reported in the scientific literature [5,24].

The mutual correlation between IR, inflammation, and oxidative stress, as well as their implication in MASLD pathogenesis [8,9], constitutes the scientific rationale of the FLAME index. The entity of both glucosidic (elevated HbA1c) and lipidic profile alterations (lower HDL) influences the worsening of hepatic fibrosis, in a complex scenario where the local and systemic inflammation, simultaneously with oxidative stress, represents a key driver for both the IR-related metabolic dysfunction hallmarks and liver fibrosis onset and worsening [25,26]. Moreover, HDL particles play a crucial role in anti-oxidative stress and are key regulators of inflammation [27,28]. Like a piece of a mosaic fitting perfectly, the alphaGST levels were included as an expression of hepatic oxidative stress [1], considering the close relationship of stress unbalance with inflammation and IR [8,9].

Relevantly, the FLAME score algorithm, compared to alphaGST alone, presented even better diagnostic power in predicting AF in the case of a total score >5 (BCO > 5), both in TrC and VlC.

A common weakness point referred to the greater part of liver fibrosis serum markers is their good NPV but fair PPV determined a lot of false positive cases. In this context, NFS and BARD scores are specifically used [29,30]. By matching age, FPG level, PLT, albumin, and AST/ALT ratio, the NFS is largely used to detect mild fibrosis (F0–2) by using the <−1.455 cut-off, NPV of 93% and the value >0.675 as a predictor of significant fibrosis (≥F3), PPV of 90% with a wide range of indeterminate values between −1.455 and 0.675, AUC: 0.88 (95% CI 0.85–0.92) [29].

Regarding BARD score, based on BMI, AST/ALT ratio, and presence of diabetes, a value between 2 and 4 was related, in a retrospectively analyzed cohort of 827 patients, with advanced fibrosis, AUC value of 0.81, and NPV of 96% [30]. Consistently, for the low cut-off of <1.45, the AUC value to detect Ishak stage 4–6 was 0.765, sensitivity 70%, and NPV 90%; for the high cut-off >3.25, the PPV was 65% and specificity 97%, with dropped accuracy for values in between [31].

A comparison study on a cohort of biopsy-proven NAFLD demonstrated good accuracy of Vibration-controlled transient elastography (VCTE) and FibroMeter in noninvasively predicting liver fibrosis with AUC of 0.84 and 0.79 for F ≥ 2, 0.83, and 0.82 for F ≥ 3, 0.86 and 0.82 for F4, respectively [32].

In this sense, a stepwise approach was proposed by Petta et al., highlighting an improved performance for liver fibrosis prediction by using initially NFS or FIB-4, followed by VCTE evaluation [33]. By using this combination, a significant decrease in incorrectly classified patients in comparison to the “single test approach” was observed, even if the rate of discordant results remained over 50% [33].

In our setting (TrC), the alphaGST alone and in combination with the other markers composing the FLAME algorithm, showed in a diagnostic comparison with NFS, FIB-4, and BARD, better performance in predicting AF, overcoming thus the dropping diagnostic accuracy of the compared scores. Moreover, by reaching the compromise of a high NPV with an equally accurate PPV, it solved the high rate of false positives resulting from using the currently applied composite scores. Relevantly, the elevated accuracy of alphaGST and FLAME, as well as the relative superiority in comparison to the other non-invasive tools, was confirmed in the VlC.

A plethora of emerging findings suggest the association between MASLD-related liver fibrosis and atherosclerotic cardiovascular events [12,13]. However, in the general population, the role of non-invasive markers of AF in predicting ACEs has been only recently and partially evaluated in the NAFLD context. In this sense, related to the available tools, no evidence of BARD accuracy in ACEs ’prediction exists and the LSM has been generally studied as a predictor of complications development, not particularly referred to ACEs, in NAFLD [34].

Schonmann et al. first reported an independent positive association between non-invasively assessed liver fibrosis by using FIB-4 and ten-year incidence of ACEs in a large cohort of the general population in Israel, suggesting the potential relevance of fibrosis markers in primary-care risk assessment [13]. Consistently, in two meta-analyses, high values of both FIB-4 and NFS were significantly associated with an increased risk (FIB-4, HR: 1.75; NFS, HR: 1.92) of cardiovascular events in NAFLD individuals [35,36].

Contrariwise, a recent large-cohort longitudinal study denied this association and revealed a not significantly different IRR of AMI, TIA, and IC, in NAFLD patients, with elevated FIB-4 in comparison to individuals with a low score [37].

For this purpose, Delgado et al. did not observe an independent association of FIB-4 or NFS with ACEs in NAFLD patients with coexisting coronary artery disease (CAD) suggesting their useless application for primary prevention of cardiovascular events in the population with underlying medium-to-high cardiovascular risk [38].

In light of this heterogeneous evidence, the availability in clinical practice of a non-invasive tool able to predict a short-term ACE incidence, independently from the other cardiovascular risk factors, appears to be crucial in the MASLD setting. For this purpose, considering that the FLAME index composing variables also explores the different components of MS, we evaluated its accuracy in predicting the first ACE (AMI, SCA, IC, or TIA) in MASLD ACE-naive patients. ROC analysis, by identifying the cut-off > 6, revealed an elevated FLAME index accuracy in predicting this outcome (AUC: 0.92, PPV:88.5%, NPV: 76%) and its better and independent predictive performance in this sense compared to the other liver fibrosis non-invasive scores (NFS AUC: 0.76; FIB-4 AUC: 0.79; BARD AUC: 0.80; LSM AUC: 0.80], hematochemical [HDL, HbA1c, triglycerides] and anthropometric [SBP, DBP, BMI] parameters. Consistently, the IRR of ACEs in MASLD patients having a FLAME > 6 was significantly higher [HR: 2.624], confirming thus the elevated predictivity of this score in our setting. The elevated FLAME performance in predicting the first ACE was also confirmed in the VlC.

Finally, as an ancillary study outcome, given the strong well-evidenced high correlation between worsening liver fibrosis and LREs’occurrence [39,40], we evaluated FLAME index performance, revealing, consistently, with constituting variables and scientific rationale, our novel score similar accuracy compared with the other non-invasive tools (LSM, BARD, FIB-4, and NFS), even in VlC. Unfortunately, since the evidence on potentially disease-modifying drugs preventing LREs in compensated cirrhotic patients was only emerging at the time of the enrollment [41], no data regarding the baseline or ongoing administration of non-selective beta-blockers (propranolol and carvedilol) were collected. Certainly, this represented a limitation of this study, although related to a merely ancillary objective.

On the other hand, focusing instead on the main study outcomes, the monocentric reported observations including a relatively limited number of patients without an external VlC represented the principal limitations of the present research. Anyway, to maintain high data reliability, the cross-validation was performed at two different and independent intervals of time, obtaining in VlC the confirmation of the results observed in TrC. In this sense, by investigating novel (alphaGST role in MASLD) and unexplored findings (performance of alphaGST and FLAME in predicting AF and ACEs in MASLD), the presented work appears as pioneering in opening the doors to replicate similar observations in larger cohorts, even followed for longer periods.

## 4. Materials and Methods

### 4.1. Experimental Design

In this prospective observational study, between January 2016 and December 2017, we consecutively enrolled patients (Training Cohort) (TrC) affected by ultrasonography (US)-detectable bright liver presenting concomitantly with the MASLD diagnostic features [5], and a group of healthy controls. The healthy controls were individuals without evidence of bright liver revealed by US examinations received within 12 months before enrollment with imaging available in personal electronic medical records (EMRs).

As a Validation Cohort (VlC), between January 2018 and May 2019, 60 MASLD patients were consecutively enrolled. At the time of enrollment, the liver steatosis was confirmed, and the entity was analytically defined by performing the controlled attenuation parameter (CAP) assessment [42]. The Alcohol Use Disorders Identification Test (AUDIT-C) questionnaire [43] assessed alcohol consumption, to preventively exclude patients potentially affected by alcoholic liver disease (ALD) from the recruitment.

As specified below, anthropometrical and clinical data were collected at the enrollment. Moreover, a 10 mL venous blood sample (5 mL of serum and 5 mL of plasma) was collected to assess biochemical parameters and alphaGST levels. MASLD patients received a non-invasive evaluation of the baseline liver disease severity by performing Liver Stiffness measurement (LSM) [44], NFS [29], FIB-4 [31], and BARD [30] scores evaluations, as well as a US-guided percutaneous liver biopsy (pLB) for diagnostic/staging purposes [45,46].

The baseline collected biochemical variables, relative to specific domains, significantly associated with AF in Trc configured the FLAME index, and, for all MASLD patients, both in TrC and VlC, the FLAME index was subsequently calculated (see the following dedicated section).

MASLD patients (TrC and VlC) were semiannually followed up over 5 years.

**Figure 6:**
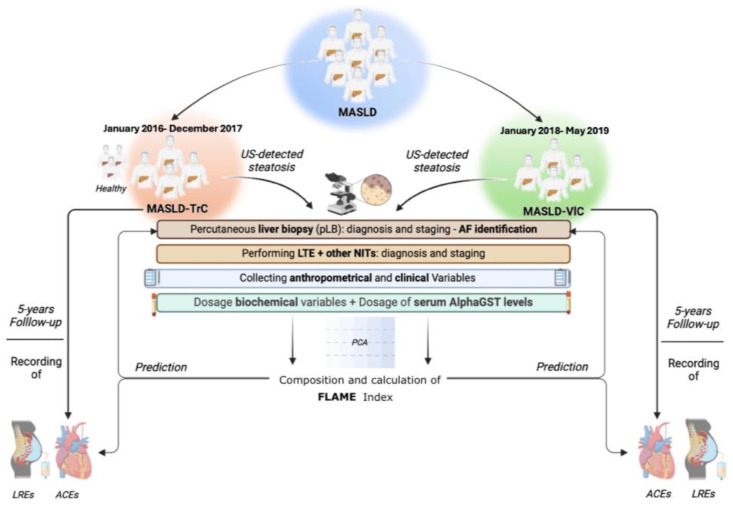
Experimental design. Anthropometrical variables included: body mass index (BMI), waist-to-hip ratio (Whr), systolic blood pressure (SBP), and diastolic blood pressure (DBP). Clinical evaluation included comorbidities, drug administration, and nutritional habits. Biochemical variables included: aspartate aminotransferase (AST), alanine aminotransferase (ALT); gamma-glutamyl transferase (GGT), alkaline phosphatase (ALP), total bilirubin (TB), platelets count (PLT), prothrombin time (PT), total cholesterol (TC); high-density lipoprotein cholesterol (HDL); low-density lipoprotein cholesterol (LDL), triglycerides (TG), plasma albumin (PA), C-reactive protein (CRP), fasting plasma glucose (FPG), and glycosylated hemoglobin (HbA1c). Alpha-Glutathione-S-transferase (AlphaGST) serum levels were assessed for each patient. Liver Transient Elastography (LTE) was adopted to assess Liver Stiffness Measurement (LSM) and Controlled Attenuation Parameter (CAP). NITs included: NFS, FIB-4, and BARD. The NAS and the Ishak score were adopted for histological staging. ACEs were: Acute Myocardial Infarction (IMA), Acute Coronary Syndrome (ACS), Ictus cerebri (IC), and Transient Ischemic Attack (TIA). LREs were: ascites, hepatic encephalopathy, gastroesophageal bleeding, jaundice, and HCC. NITs: Noninvasive tools. US: ultrasound; NFS: NAFLD Fibrosis score; FIB-4: Fibrosis-4; BARD: BMI-AST/ALT Ratio-Diabetes; NAS: NAFLD activity score. ACE: Acute Cardiovascular Event; LRE: Liver Related Event.

During the follow-up period, for all MASLD patients, the first Liver-related event (LRE) occurrence was registered, whereas, limitedly to ACE-naïve MASLD individuals, the occurrence of the first ACE was recorded. LREs were defined by ascites, hepatic encephalopathy, gastroesophageal bleeding, jaundice in not cholestatic-related liver disorders, and HCC [39]. ACEs were: (1) Acute Myocardial Infarction (IMA), (2) Acute Coronary Syndrome (ACS), (3) Ictus cerebri (IC), and (4) Transient Ischemic Attack (TIA) [47]. Furthermore, during the observation period, on follow-up visits, patients received a screening endoscopy for esophageal varices (OEVs) according to the guidelines available at that time [48]; based on this, the onset of clinically significant portal hypertension (CSPH) was definable by the evidence of OEVs [48]. The experimental design is reported in Figure 6.

The accuracy of the alphaGST blood levels and the FLAME index in the prediction of AF in comparison to the currently available non-invasive composite tools for MASLD patients (NFS, FIB-4, BARD score, and LSM) was identified as the primary study outcome.

The accuracy of the FLAME index in the prediction of the 5 years first ACE occurrence in ACE-naïve MASLD individuals, in comparison to the hematochemical (High-density lipoprotein, triglycerides, glycosylated Hemoglobin), anthropometrical parameters (Body Mass Index- BMI, systolic blood pressure, diastolic blood pressure) [49], and other non-invasive composite tools for liver fibrosis (NFS, FIB-4, BARD score, and LSM) [11], was identified as the secondary outcome.

The evaluation of FLAME index’s accuracy in the prediction of the first LRE in MASLD individuals, in comparison to the currently available non-invasive composite tools for MASLD patients (NFS, FIB-4, BARD score, and LSM) represented an ancillary study outcome.

The entire study protocol was registered on the NIH U.S. National Library of Medicine database of clinical trials and is available online at https://www.clinicaltrials.gov (accessed on 9 September 2024) (NCT05804955). All authors had access to the study data and reviewed and approved the final manuscript.

### 4.2. Patients

This study complies with the Declaration of Helsinki (1975) and has been approved by the ethical committee of the University of Campania Luigi Vanvitelli in Naples (prot. n. 531/2016).

In the present study (Figure 1), after signing the informed consent, we consecutively enrolled healthy subjects (i.e., individuals without previous US evidence of bright liver) as the control group and a TrC of patients affected by US-detectable bright liver contemporary presenting the following MASLD diagnostic features: overweight or obesity, defined as BMI > 25 kg/m^2^, or type 2 diabetes mellitus (T2DM) or presence of ≥one of the following criteria: (A) waist circumference ≥ 94 cm (males) and ≥80 cm (females); (B) blood pressure ≥ 130/85 mmHg or specific drug treatment; (C) plasma triglycerides (TG) ≥ 150 mg/dL or specific drug treatment; (D) plasma high-density lipoprotein (HDL) cholesterol < 40 mg/dL (males) and <50 mg/dL (females) or specific drug treatment; (E) fasting plasma glucose (FPG): 100–125 mg/dL or 2 h post-load glucose levels 140–199 mg/dL or glycated hemoglobin (HbA1c) > 5.7% [5].

As a VlC, after signing the informed consent, patients with US-detectable bright liver presenting the previously reported MASLD features [5] were consecutively enrolled (VlC), receiving a pLB.

At the moment of enrollment, all MASLD patients were classifiable as “NAFLD”, presenting one or more of the anthropometrical, biochemical, and clinical manifestations of the MS [5,50].

The enrollment was carried out at the Hepato-Gastroenterology Division of the University of Campania Luigi Vanvitelli in the interval from January 2016 to December 2017 (TrC) and January 2018 to May 2019 (VlC). The inclusion criteria were age between 18 and 80 years and MASLD diagnosis. Exclusion criteria were the presence of chronic inflammatory diseases, acute or chronic kidney diseases, rheumatoid arthritis, systemic lupus erythematosus, or other major systemic inflammatory diseases or tumors, ongoing infections, alcohol or drug abuse history, other etiologies of chronic liver damage, previous HCC diagnosis, use of hepatoprotective drugs, decompensated liver cirrhosis (Child-Pugh B and Child-Pugh C) at the moment of the enrollment or in the previous 12 months, and psychological/psychiatric problems that could have invalidated the informed consent.

MASLD patients were followed up every six months to detect ACEs (ACE-naïve MASLD patients) and LREs (all MASLD patients) occurrence.

### 4.3. Anthropometrical, Clinical, and Nutritional Assessment

The collection of anthropometrical parameters included the determination of BMI by dividing the weight by the square of height (kg/m^2^), directly measured waist-to-hip ratio (Whr), SBP (mmHg), and diastolic blood pressure (DBP) (mmHg). 

Clinical evaluation included the collection of a complete medical history and the assessment of alcohol consumption, nutritional habits (including physical exercise and dietetic attitudes), smoking, drug abuse, and the baseline and follow-up therapy record (including, among others, antiaggregant/anticoagulant medications, statins, and glucagon-like peptide-1 receptor agonist-GLP1RA administration) for each patient (Supplementary Table S4). 

To assess physical exercise, at enrollment, we submitted a specific medical-assisted questionnaire composed of a few questions according to the current clinical practice guidelines (CPGs) [46] (Supplementary Table S5). Compliance with the Mediterranean diet regimen was assessed at enrollment by submitting the medical-assisted questionnaire “Mediterranean diet score” [51] (Supplementary Table S6).

### 4.4. Biochemical Assessment

The evaluated biochemical data were: aspartate aminotransferase (AST), alanine aminotransferase (ALT), gamma-glutamyl transferase (GGT), alkaline phosphatase (ALP), total bilirubin (TB), platelet count (PLT), plasma albumin (PA), prothrombin time (PT) total cholesterol (TC), HDL, Low-density lipoprotein (LDL) cholesterol, TG, C-reactive protein (CRP), insulin (μU/mL), FPG, and glycosylated hemoglobin (HbA1c) (%). Insulin, GGT, and CRP levels were measured enzymatically using commercially available kits (R&D Systems, Minneapolis, MN), AST, ALT, and glucose using a colorimetric assay kit (Amplite 13801/13803 and Thermo Fisher Scientific EIAGLUC). 

The homeostatic model assessment for insulin resistance (HOMA-IR) was calculated by using the validated formula: fasting insulin (μU/mL) × FPG (mmol/L)/22.5 [52].

### 4.5. AlphaGST Levels Assessment

Plasma was stored frozen at −20 °C until assayed. The protein level of alphaGST (pg/mL) was determined on all frozen plasma samples by using the specific human enzyme-linked immunosorbent assay (ELISA) kit “HEPKIT” (Biotrin International, Dublin, Ireland), according to the manufacturer instructions provided. All the measurements were performed in duplicate for each patient.

### 4.6. Abdominal US, Liver Stiffness Measurement, and Controlled Attenuation Parameter Assessment

Ultrasonography B-mode evaluation performed by an expert physician was used to identify the presence of a bright liver. The brightness of the liver parenchyma, by comparing the liver and kidney echogenicity ratio, together with the appearance of hepatic veins and the diaphragm, was used for this scope. The ultrasound machinery used was the GE Logiq E10^®^. LSM was performed by using FibroScan^®^ [version 502 (Echosens, Paris, France)] with M and XL probes [53]. We decided to use the XL probe when the ultrasound measured distance between the skin and the liver capsule resulted in greater than 2.5 cm and/or when the patient’s BMI was >30.

The FibroScan^®^ was performed by an expert physician obtaining 10 acceptable measurements (defined as successful LSM). The criteria proposed by Boursier et al. were used to consider the measurement “very reliable” (IQR/M ≤ 0.1), “reliable” (0:1 < IQR/M ≤ 0.3 or IQR/M > 0:3 with LS median < 7.1 kilopascal), or “poorly reliable” (IQR/M > 0.3 with LS median ≥ 7.1 kilopascal (kPa) [53,54].

The following LSM cut-off scores were used to identify the different liver fibrosis stages according to the Metavir score: (a) F0–F2 ≤ 9.6 kPa; (b) F3: 9.7–13.5 kPa; (c) F4 ≥ 13.6 kPa [55].

The entity of liver steatosis was analytically assessed by using CAP. The CAP measures ultrasonic attenuation in the liver at 3.5 MHz using signals acquired by the FibroScan^®^ M and XL probes based on physical principles described elsewhere [54,56]. The CAP was measured only on validated measurements according to the same criteria used for LSM [53,54,56]. In detail, based on the CAP scores, the enrolled patients were stratified as follows: S0, no steatosis (0–10% fat; 0–237 dB/m); S1, mild steatosis (11–33% fat; 238–259 dB/m); S2, moderate steatosis (34–66% fat; 260–292 dB/m); and S3, severe steatosis (>67% fat; ≥293 dB/m) following the calculation of the attenuation of ultrasonic signals used for Liver Transient Elastography (LTE) [53,54,56].

### 4.7. Antropobiochemical Non-Invasive Liver Fibrosis Assessment Tools

NFS was determined by using the formula: −1.675 + 0.037 × age (years) + 0.094 × BMI (kg/m^2^) + 1.13 × diabetes (yes = 1, no = 0) + 0.99 × AST/ALT ratio − 0.013 × PLT (x109/L) − 0.66 × PA (g/dL) [57]. FIB-4 index was calculated as follows: (Age × AST)/(PLT) × √(ALT) [30]. The BARD scoring system was assessed according to Harrison et al.: BMI ≥ 28 = 1 point, AST/ALT ratio ≥ 0.8 = 2 points, and type 2 diabetes mellitus = 1 point [30].

### 4.8. Ultrasound-Guided Percutaneous Liver Biopsy and Histological Assessment

All MASLD patients received a US-guided pLB for diagnostic/staging purposes. By using a 19-gauge tru-cut needle, an expert physician obtained the liver sample, stored it immediately in formalin, and sent it afterward to the pathologists for histological analysis.

The suitability of the sample was defined by the presence of at least 15 portal spaces and the lack of this characteristic was followed by a resampling.

The MASH diagnosis was made according to standard histopathologic criteria, and the NAFLD activity score (NAS) established by Kleiner, as the sum of steatosis [0–3], lobular inflammation [0–3], and hepatocellular ballooning [0–2] degrees [58]. A NAS > 5 defined steatohepatitis [58].

The fibrosis was staged by using the Ishak score (stages 0–6) as follows: stage 0 (normal liver); stage 1 (fibrosis expansion of a few portal tracts); stage 2 (fibrosis of all portal tracts); stage 3 (fibrous expansion of most portal areas with occasional portal-to-portal bridges); stage 4 (fibrous expansion of portal areas with marked bridges); stage 5 (marked bridges with occasional nodules); stage 6 (established cirrhosis with the tissue entirely composed of nodules [59]. According to the Metavir score, patients presenting an Ishak stage 0–3, 4–5, and 6 were, respectively, considered as affected by F0–F2, F3, and F4 [60].

### 4.9. Statistical Analysis

Continuous data were described as mean and standard deviations, while categorical variables as n (%). The Kolmogorov–Smirnov test for normality was performed to evaluate if the parametric or non-parametric analysis should be applied. Mann–Whitney and *t*-test for independent groups, the Kruskal–Wallis test, or ANOVA test with post hoc Tukey analysis, in the case of non-normal or normal distribution, respectively, were performed to compare the continuous variables. Linear regression analysis was adopted to evaluate the relationships (R) between continuous variables.

Principal component analysis (PCA) was adopted to adequately select (eigenvalues > 1.5) the biochemical variables composing the three dimensions: “Free plasma glucose/insulin resistance-related abnormalities” (FPG, Insulin levels, HOMA-IR, HbA1c), “Lipid-Associated Metabolic alterations” (TC, HDL, LDL, TG), and “Excretion of liver-injuring toxic metabolites/anti-oxidative stress mechanisms—impairment” (AST, ALT, AlphaGST, GGT, ALP) (Principal Component) (PC).

ROC analysis with the Youden index calculation for the identification of BCO values determined the thresholds to consider for each PCA-selected variable. ROC analysis with the Youden index calculation for the identification of BCO values, integrally with the Chi-Square test for the sensitivity, specificity, negative predictive value (NPV), and positive predictive value (PPV) evaluation, was also performed to estimate the accuracy of alphaGST levels, FLAME index, LSM, NFS, FIB-4, and BARD score in the prediction of AF and 5 years LREs occurrence in all MASLD individuals, as well as the accuracy of the FLAME index in comparison to hematochemical (HDL, TG, HbA1c) variables, anthropometrical parameters (BMI, SBP, DBP), and other non-invasive composite tools for liver fibrosis (NFS, FIB-4, BARD score, and LSM) in the prediction of 5 years first ACE occurrence in ACE-naïve MASLD patients.

The odds ratios (ORs) of the study variables on these events were obtained by using multinomial logistic regression models, considering the confounding variables [sex, age, BMI, diabetes, arterial hypertension, Mediterranean diet compliance, physical exercise, smoking, drug administration (including anticoagulant/antiaggregant, statins, and GLP1-RA), and alcohol intake]. Time-to-event analyses on ACEs occurrence upper and under the FLAME index ROC-analysis identified BCO was performed using the Kaplan–Meier method and the Log-rank test for the curve comparison considering a *p*-value < 0.05 as statistically significant.

The Standards for Reporting of Diagnostic Accuracy Studies (STARD) flowchart relative to TrC is completely reported in Supplementary Figure S4.

Statistical significance was defined as *p* < 0.05 in a two-tailed test with a 95% confidence interval (C.I.). Statistical Program for Social Sciences (SPSS^®^) vs.18.0 was used to perform the analysis.

## 5. Conclusions

In the MASLD hepatologic era, the identification of non-invasive markers to early identify the AF represents a crucial challenge in the liver-focused research and clinical field. In this scenario, the alphaGST and the FLAME index appear as useful tools in the prediction of disease impairment in MASLD patients. For such individuals, in parallel to the worsening of liver fibrosis, the occurrence of atherosclerotic-related ACEs currently constitutes the first cause of death. Therefore, the availability of scores able also to identify individuals at ACEs-higher risk may help clinicians in the routinary management of MASLD patients.

## Figures and Tables

**Table 1:** Demographic, anthropometric, biochemical data, and non-invasive tools of the study population (Healthy, Training Cohort-TrC, and Validation Cohort-VlC).

**Demographic data**					
	Healthy (n:30) (A)	TrC-MASLD (n: 200) (B)	VlC-MASLD (n: 60) (C)	*p*-value A vs. B	*p*-value B vs. C
Gender-Male (n and %)	13 (43%)	119 (59%)	34 (56.7%)	n.s. *	n.s. *
Age (mean ± SD)	49.97 ± 9.89	59 ± 12.52	59.77 ± 12.89	n.s. **	n.s. **
**Anthropometric indexes**					
Variables (mean ± SD)	Healthy (A)	TrC-MASLD (B)	VlC-MASLD (C)	*p*-value ** A vs. B	*p*-value ** B vs. C
BMI (Kg/m^2^)	24.97 ± 2.17	32.13 ± 2.79	31.37 ± 3.31	**<0.0001**	n.s. **
WhR	0.81 ± 0.05	1.42 ± 0.68	1.28 ± 0.91	**<0.0001**	n.s. **
SBP (mmHg)	115.3 ± 9.73	131.1 ± 12.31	130 ± 10.85	**<0.0001**	n.s. **
DBP (mmHg)	74.67 ± 10.42	87.45 ± 7.91	87.50 ± 8.26	**<0.0001**	n.s. **
**Biochemical parameters**					
Variables (mean ± SD)	Healthy (A)	TrC-MASLD (B)	VlC-MASLD (C)	*p*-value ** A vs. B	*p*-value ** B vs. C
AST (IU/L)	31.30 ± 10.14	50.86 ± 29.18	50.08 ± 26.26	**<0.0001**	n.s. **
ALT (IU/L)	39.37 ± 17.57	59.89 ± 30.36	59.65 ± 32.59	**<0.0001**	n.s. **
GGT (IU/L)	40.57 ± 17.99	74.22 ± 45.94	72.88 ± 47.20	**<0.0001**	n.s. **
ALP (IU/L)	92.33 ± 51.75	91.92 ± 22.38	93.89 ± 20.81	**0.031**	n.s. **
PLT (mm^3^)	327.06 ± 58.92	236.7 ± 15.05	247.9 ± 13.06	**<0.0001**	n.s. **
TC (mg/dL)	135.2 ± 42.07	172.7 ± 46.96	169.2 ± 51.41	**<0.0001**	n.s. **
LDL (mg/dL)	95.93 ± 27.29	120.4 ± 37.19	120.8 ± 41.9	**0.0002**	n.s. **
HDL (mg/dL)	44.73 ± 9.67	41.92 ± 9.34	42.07 ± 8.79	n.s.	n.s. **
TG (mg/dL)	109.5 ± 32.14	141.8 ± 60.58	142.5 ± 54.34	**0.003**	n.s. **
FPG (mg/dL)	100.7 ± 9.35	121.3 ± 16.74	120.2 ± 15.92	**<0.0001**	n.s. **
Insulin (μU/mL)	7.03 ± 1.62	12.19 ± 3.42	11.63 ± 3.27	**<0.0001**	n.s. **
HbA1c (%)	4.01 ± 0.42	5.24 ± 1.45	5.38 ± 1.19	**<0.0001**	n.s. **
HOMA-IR	1.77 ± 0.54	3.55 ± 1.25	3.28 ± 1.53	**<0.0001**	n.s. **
TB (mg/dL)	0.94 ± 0.17	1.52 ± 1.09	1.65 ± 1.18	n.s.	n.s. **
Albumin (g/L)	4.42 ± 0.29	4.05 ± 0.52	3.94 ± 0.52	**<0.0001**	n.s. **
PT (seconds)	9.93 ± 7.42	99.94 ± 7.83	101.1 ± 8.85	n.s.	n.s. **
C-RP (mg/L)	0.61 ± 0.20	1.63 ± 0.75	1.64 ± 0.34	**<0.0001**	n.s. **
AlphaGST (pg/mL)	1695 ± 191.10	3828 ± 236.10	3821 ± 239.6	**<0.0001**	n.s. **
**Non-invasive tools for liver disease severity assessment**					
Variables (mean ± SD)	Healthy (A)	TrC-MASLD (B)	VlC-MASLD (C)	*p*-value ** A vs. B	*p*-value ** B vs. C
LSM (kPa)	/	9.67 ± 4.91	10.34 ± 4.68	/	n.s. **
CAP (dB/m)	/	271.3 ± 11.69	270.2 ± 11.97	/	n.s. **
NFS	/	−0.89 ± 2.04	−0.87 ± 1.98	/	n.s. **
FIB-4	/	2.24 ± 1.72	2.32 ± 1.83	/	n.s. **
BARD	/	2.17 ± 1.15	2.21 ± 1.16	/	n.s. **
**Nutritional Habits**					
	Healthy (n:30) (A)	TrC-MASLD (n:200) (B)	VlC-MASLD (n: 60) (C)	*p*-value A vs. B	*p*-value B vs. C
MedDiet (n and %)	12 (40%)	91 (45.5%)	26 (43.3%)	n.s. *	n.s. *
Active exercise (n and %)	13 (43.3%)	88 (44%)	28 (46.6%)	n.s. *	n.s. *

BMI: Body mass index, WhR: waist to hip ratio, AST: aspartate aminotransferase, ALT: alanine aminotransferase, GGT: gamma-glutamyl transferase, ALP: alkaline phosphatase, PLT: platelets count, PT: prothrombin time, HDL: high-density lipoprotein, LDL: low-density lipoprotein, FPG: fasting plasma glucose, HOMA-IR: homeostatic model assessment for insulin resistance; HbA1c: glycosylated hemoglobin, TG: triglycerides, TC: total cholesterol, TB: total bilirubin, LSM: liver stiffness measurement, CAP: controlled attenuation parameter, NFS: NAFLD fibrosis score, FIB-4: Fibrosis-4, BARD: BMI-AST/ALT Ratio-Diabetes, AlphaGST: Alpha-glutathione-S-transferase, SD: standard deviation. TrC: training cohort, VlC: Validation Cohort; n: number. ** Mann–Whitney U test. * Chi-square test. Statistically significant differences (*p* < 0.05) are reported in bold; n.s.: not statistically significant.

**Table 2:** Scoring calculation system of the FLAME index.

	**Domain**	**Variable**	**Threshold**	**Points**
A	Free plasma glucose/insulin resistance-related abnormalities	HbA1c (%)	≤5.5	1
			>5.5	2
B	Lipid- Associated Metabolic alterations	HDL (mg/dL)	HDL > 43.5	1
			HDL ≥ 43.5	2
C	Excretion of liver-injuring toxic metabolites/anti-oxidative stress mechanisms—impairment	AlphaGST (pg/mL)	≤3917	2
			>3917	4
FLAME TOTAL SCORE (min–max)			MIN: 4-MAX: 8	

HDL: High-density lipoprotein cholesterol; alphaGST: Alpha-glutathione-S-transferase.

## Data Availability

The data that support the findings of this study are available from the corresponding author upon reasonable request.

## Supplementary Materials

The following supporting information can be downloaded at: https://www.mdpi.com/article/10.3390/ijms26020761/s1.

## Abbreviations

ACE (acute cardiovascular events); ACS (Acute Coronary Syndrome); AF (advanced fibrosis); ALP (alkaline phosphatase); AlphaGST (Alpha-glutathione-S-transferase); ALT (alanine aminotransferase); AST (aspartate aminotransferase); AUC (area under the ROC curve); AUDIT-C (Alcohol Use Disorders Identification Test); BARD (BMI-AST/ALT Ratio-Diabetes); BCO (best cut-off); BMI (Body Mass Index); CAD (coronary artery disease); CAP (controlled attenuation parameter); CI (confidence interval); CPGs (clinical practice guidelines); CRP (C-reactive protein); DBP (diastolic blood pressure); ELISA (Enzyme-linked immunosorbent assay); FIB-4 (Fibrosis-4); FPG (fasting plasma glucose); GGT (gamma-glutamyl transferase); GH (Glycosylated Hemoglobin); GST (glutathione-S-transferase); HbA1c (glycosylated hemoglobin); HCC (hepatocellular carcinoma); HDL (High-density lipoprotein); HOMA-IR (homeostasis model assessment for insulin resistance); HR (hazard ratio); IC (Ictus cerebri); IMA (Acute Myocardial Infarction); IQR/M (interquartile range/median); IR (insulin resistance); IRR (incidence ratio rate); pLB (percutaneous liver biopsy); LDL (Low-density lipoprotein); LTE (Liver Transient Elastography); LSM (Liver stiffness measurement); MASLD (Metabolic Dysfunction-Associated Steatotic Liver Disease); MS (metabolic syndrome); NAFLD (Non-alcoholic Fatty Liver Disease); NAS (NAFLD activity score); MASH (Metabolic Dysfunction-associated Steatohepatitis); NFS (NAFLD fibrosis score); NPV (negative predictive value); OEVs: oesophageal varices; OR (odds ratio); PA (plasma albumin); PC (Principal Component); PCA (Principal Component Analysis); PLT (platelets count); PPV (positive predictive value); PT (prothrombin time); ROC (Receiver operator characteristic); SBP (systolic blood pressure); SCA (acute coronary syndrome); SS (simple steatosis); TB (total bilirubin); TC (total cholesterol); TG (triglycerides); TIA (Transient Ischemic Attack); US (ultrasonography); VCTE (Vibration controlled transient elastography); WHR (waist-to-hip ratio).
