# Supplementary material for: FLAME: Training and Validating a Newly Conceived Model Incorporating Alpha-Glutathione-S-Transferase Serum Levels for Predicting Advanced Hepatic Fibrosis and Acute Cardiovascular Events in Metabolic Dysfunction-Associated Steatotic Liver Disease (MASLD)
Source: Int J Mol Sci. 2025 Jan 17;26(2):761. doi: 10.3390/ijms26020761 (PMC11765617; doi:10.3390/ijms26020761)
Supplement: Supplementary file 1 [file ijms-26-00761-s001.zip › Supplementary Figure S1.pdf]

A

Dimension: Free plasma glucose/insulin resistance-related abnormalities

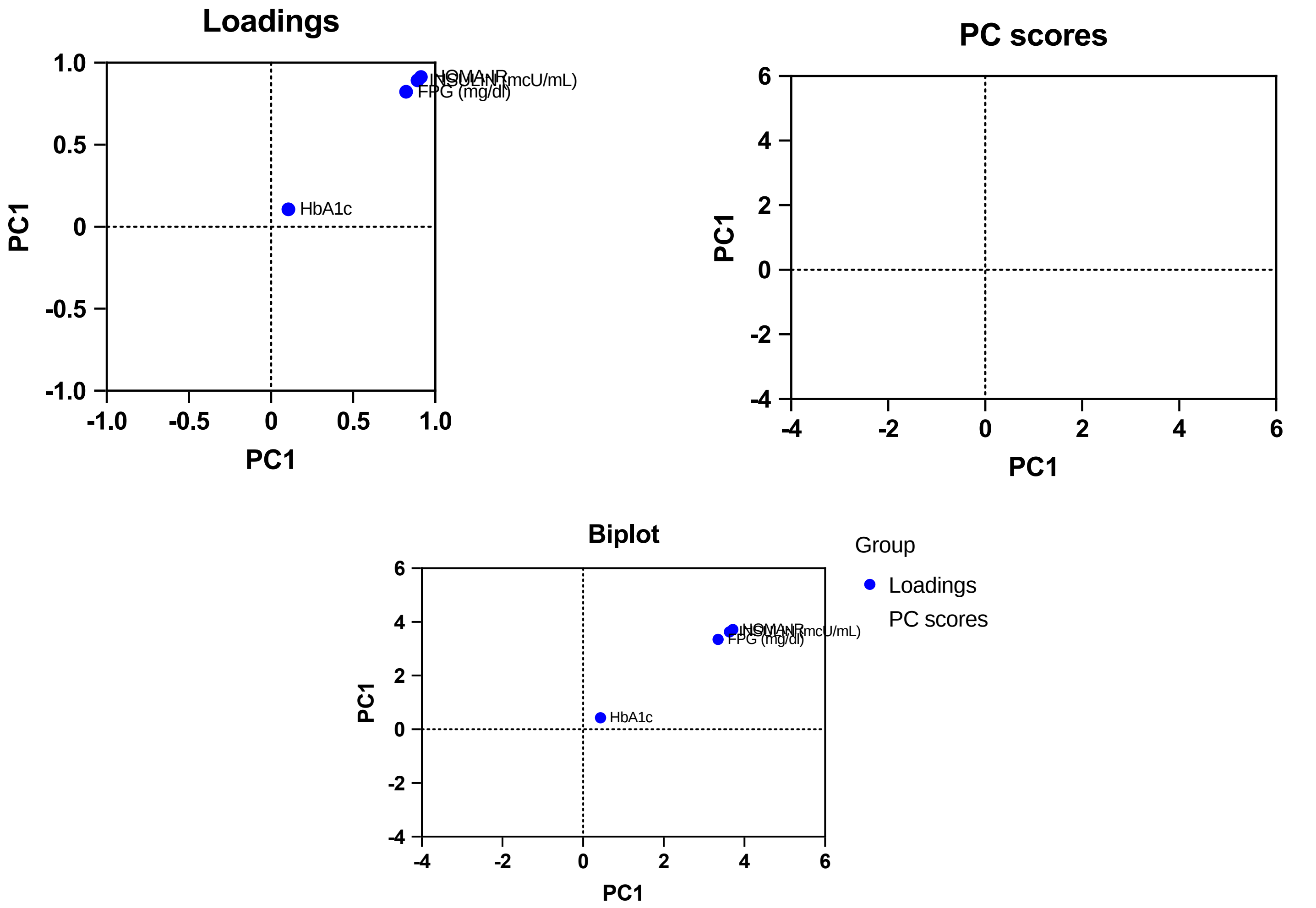

PC summary	PC1	PC2	PC3	PC4
Eigenvalue	2.316	1.021	0.4292	0.2337
Proportion of variance	57.89%	25.54%	10.73%	5.84%
Cumulative proportion of variance	57.89%	83.43%	94.16%	100.00%
Component selection	Selected			

	Eigenvalue
PC1	2.316
PC2	1.021
PC3	0.429
PC4	0.234

Var	PC1
HbA1c	0.106
FPG (mg/dl)	0.822
INSULIN (mcU/mL)	0.892
HOMA-IR	0.913

B

Dimension: Lipid-Associated Metabolic alterations

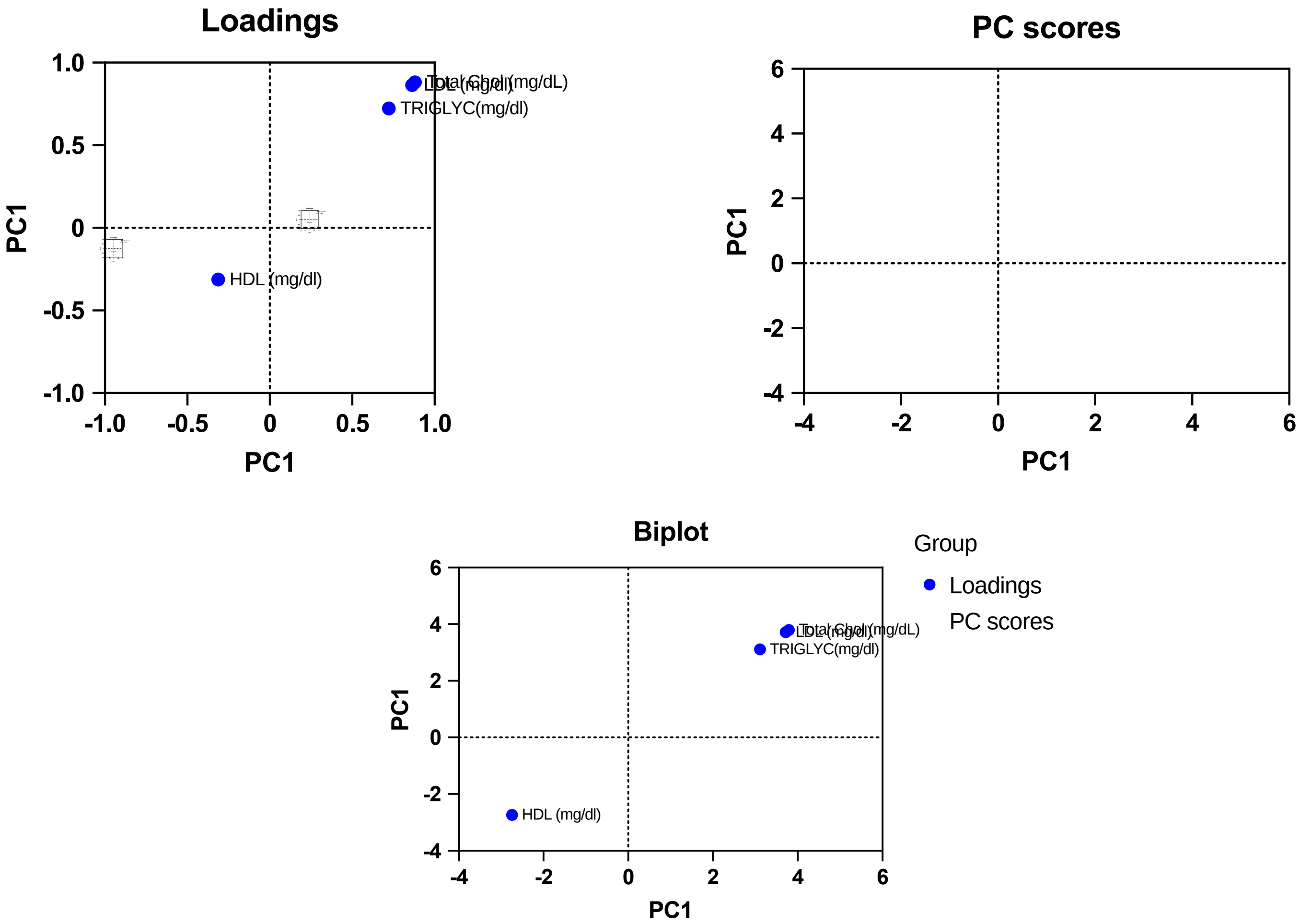

PC summary	PC1	PC2	PC3	PC4
Eigenvalue	2.140	1.072	0.6773	0.1115
Proportion of variance	53.49%	26.79%	16.93%	2.79%
Cumulative proportion of variance	53.49%	80.28%	97.21%	100.00%
Component selection	Selected			

	Eigenvalue
PC1	2.140
PC2	1.072
PC3	0.677
PC4	0.111

Var	PC1
HDL (mg/dl)	-0.314
LDL (mg/dl)	0.863
TRIGLYC(mg/dl)	0.722
Total Chol (mg/dL)	0.881

C Dimension: Excretion of liver-injuring toxic metabolites/anti-oxidative stress mechanisms - impairment

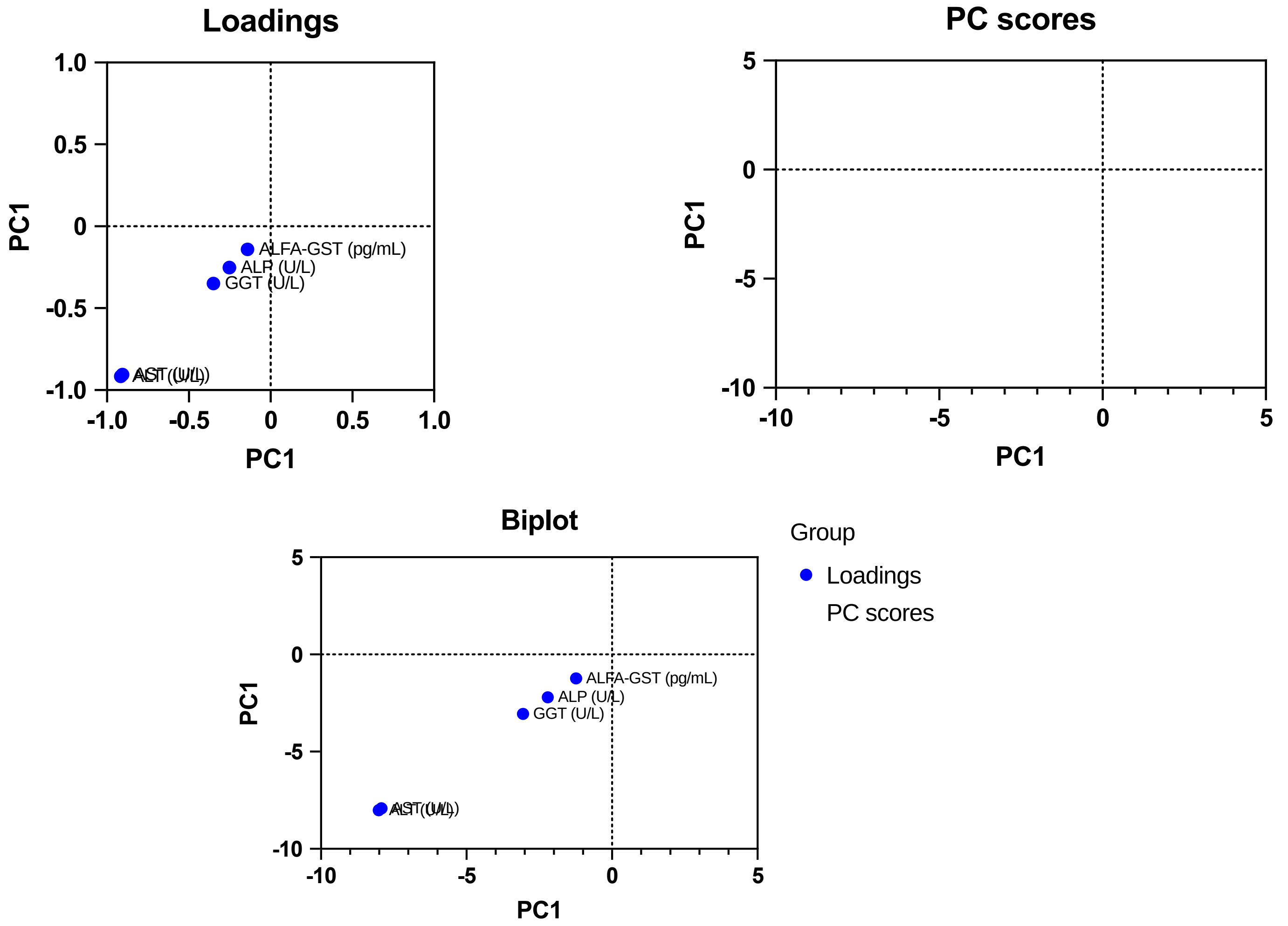

PC summary	PC1	PC2	PC3	PC4	PC5
Eigenvalue	1.869	1.292	0.9756	0.6745	0.1886
Proportion of variance	37.39%	25.84%	19.51%	13.49%	3.77%
Cumulative proportion of variance	37.39%	63.22%	82.74%	96.23%	100.00%
Component selection	Selected				

	Eigenvalue
PC1	1.869
PC2	1.292
PC3	0.976
PC4	0.675
PC5	0.189

Var	PC1
ALFA-GST (pg/mL)	-0.142
AST (U/L)	-0.906
ALT (U/L)	-0.917
GGT (U/L)	-0.350
ALP (U/L)	-0.253

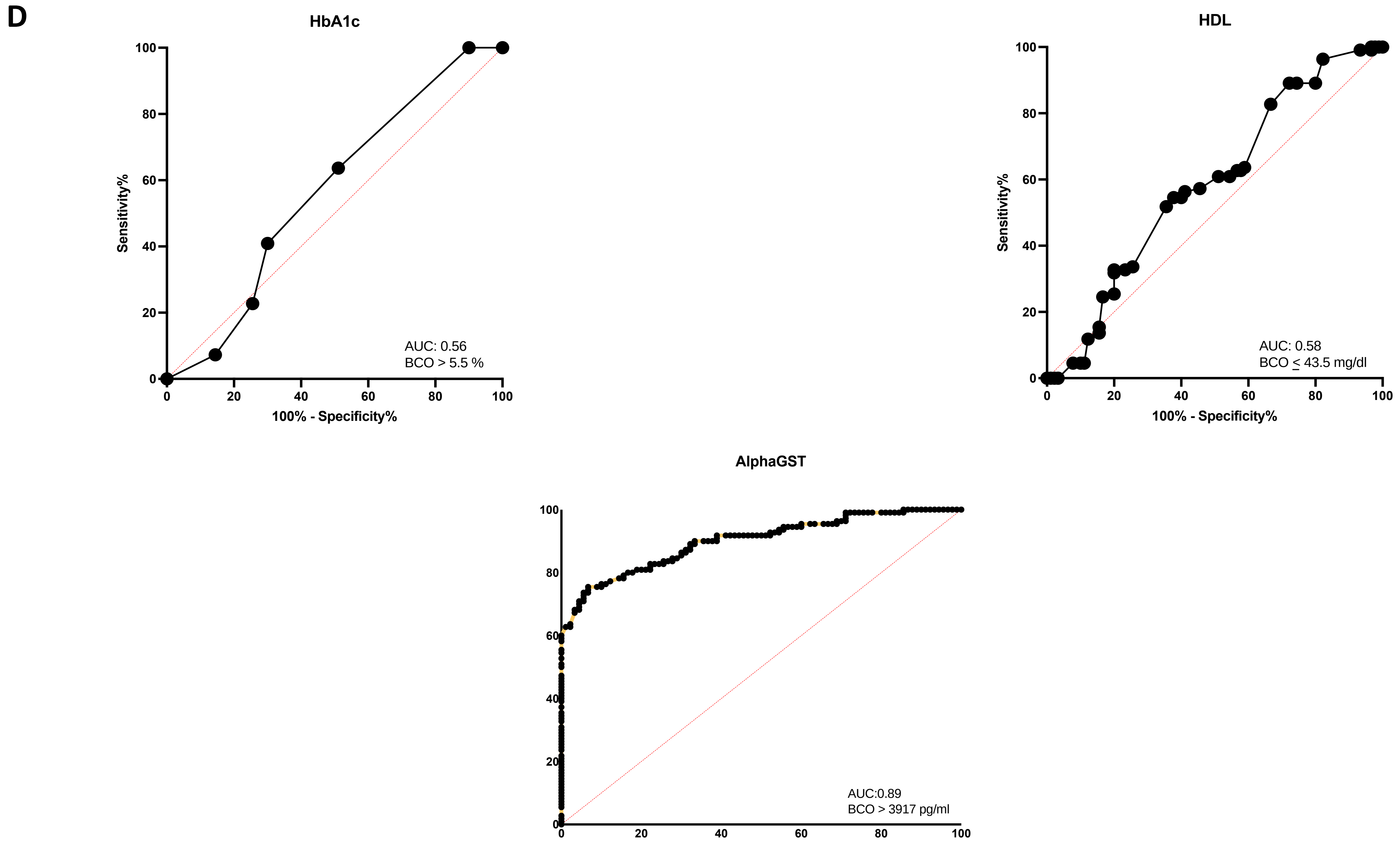
D
HbA1c
Sensitivity%
100% - Specificity%
AUC: 0.56
BCO > 5.5 %
HDL
Sensitivity%
100% - Specificity%
AUC: 0.58
BCO ≤ 43.5 mg/dl
AlphaGST
AUC:0.89
BCO > 3917 pg/ml

E

FLAME Index: scientific rationale

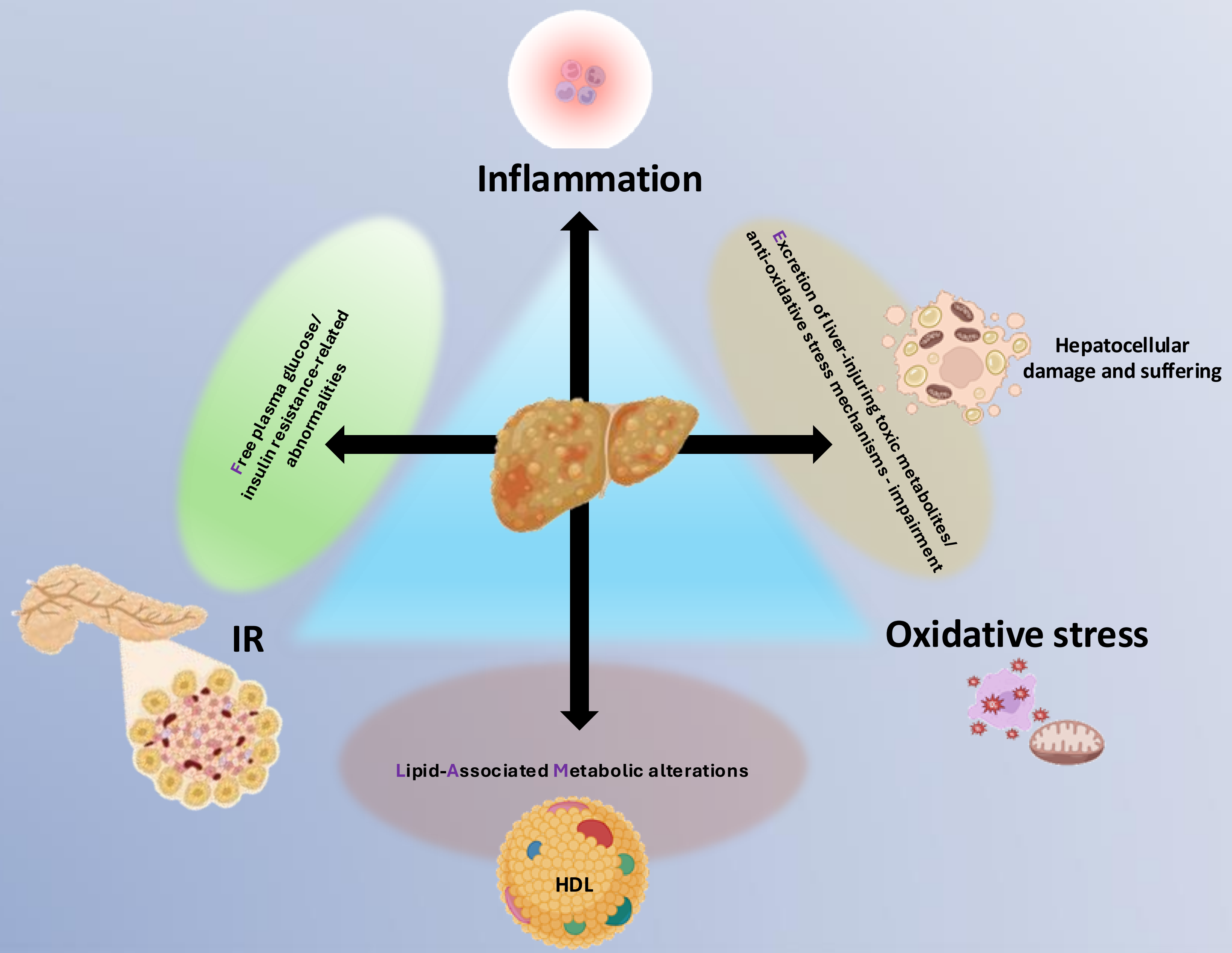

	Variable	Cut-off	Points
Free plasma glucose/insulin resistance-related abnormalities	HbA1c (%)	≤ 5.5	1
		> 5.5	2
Lipid-Associated Metabolic alterations	HDL (mg/dl)	HDL > 43.5	1
		HDL ≤ 43.5	2
Excretion of liver-injuring toxic metabolites/anti-oxidative stress mechanisms - impairment	AlphaGST (pg/ml)	≤ 3917	2
		> 3917	4
