# Supplementary material for: FLAME: Training and Validating a Newly Conceived Model Incorporating Alpha-Glutathione-S-Transferase Serum Levels for Predicting Advanced Hepatic Fibrosis and Acute Cardiovascular Events in Metabolic Dysfunction-Associated Steatotic Liver Disease (MASLD)
Source: Int J Mol Sci. 2025 Jan 17;26(2):761. doi: 10.3390/ijms26020761 (PMC11765617; doi:10.3390/ijms26020761)
Supplement: Supplementary file 1 [file ijms-26-00761-s001.zip › Supplementary Table S1.pdf]

Supplementary Table S1A. Distribution of the first Acute Cardiovascular Events according to the baseline fibrosis stage (Training Cohort).

		Fibrosis stage: F0F2	Fibrosis stage: F3	Fibrosis stage: F4	Advanced Fibrosis (F3F4)	p-value (F0-F2 vs F3F4) *
**Number and type of first ACE over 5 years**	AMI	1	6	8	14	**p<0.0001**
	ACS	1	6	13	19	**p<0.0001**
	IC	0	0	3	3	**p<0.0001**
	TIA	1	2	4	6	**p<0.0001**
	**Total**	**3**	**14**	**28**	**42**	**45**

*AMI: Acute Myocardial Infarction; ACS: Acute Coronary Syndrome; IC: Ictus cerebri: TIA: Transient ischemic attack. *Chi-square test.*

Supplementary Table S1B. Distribution of the first Acute Cardiovascular Events according to the baseline fibrosis stage (Validation Cohort).

		Fibrosis stage: F0F2	Fibrosis stage: F3	Fibrosis stage: F4	Advanced Fibrosis (F3F4)	p-value (F0-F2 vs F3F4) *
**Number and type of first ACE over 5 years**	AMI	2	1	3	4	**p<0.0001**
	ACS	1	3	4	7	**p<0.0001**
	IC	1	0	1	1	**p<0.0001**
	TIA	1	1	1	2	**p<0.0001**
	**Total**	**5**	**5**	**9**	**14**	**19**

*AMI: Acute Myocardial Infarction; ACS: Acute Coronary Syndrome; IC: Ictus cerebri: TIA: Transient ischemic attack. *Chi-square test.*
