# Supplementary material for: FLAME: Training and Validating a Newly Conceived Model Incorporating Alpha-Glutathione-S-Transferase Serum Levels for Predicting Advanced Hepatic Fibrosis and Acute Cardiovascular Events in Metabolic Dysfunction-Associated Steatotic Liver Disease (MASLD)
Source: Int J Mol Sci. 2025 Jan 17;26(2):761. doi: 10.3390/ijms26020761 (PMC11765617; doi:10.3390/ijms26020761)
Supplement: Supplementary file 1 [file ijms-26-00761-s001.zip › Supplementary Table S2 .pdf]

Supplementary Table S2. Logistic regression analysis evaluating the variables associated with the first Acute Cardiovascular Event (ACE) occurrence over 5 years in MASLD patients

**Outcome: First Acute Cardiovascular Events along 5 years of follow-up**			
**Variable**	**Odds Ratio**	**Confidence Interval (95%)**	**p-value**
BMI	1.003	0.998-1.009	0.191
Total Cholesterol (mg/dl)	0.995	0.960-1.031	0.782
HDL (mg/dl)	1.019	0.898-1.156	0.769
LDL (mg/dl)	1.000	0.960.1.041	0.993
Tryglicerides (mg/dl)	0.998	0.979-1.016	0.789
HbA1c %	1.154	0.720-1.847	0.552
SBP (mm/hg)	1.051	0.929-1.189	0.427
DBP (mm/Hg)	0.971	0.840-1.122	0.689
NFS	0.625	0.299-1.303	0.210
FIB4	0.629	0.349-1.135	0.124
BARD	1.355	0.526-1.493	0.529
LSM (kPa)	1.112	0.885-1.396	0.362
FLAME INDEX	8.375	8.260-14.253	**<.0.0001**
Age	0.971	0.906-1.041	0.406
Sex (Male:1; Female:0)	0.772	0.048-1.619	0.154
Alcohol intake > 2 Unit/ die (Yes: 1; No:0)	0.851	0.106-3.172	0.530
Smoke (Yes: 1; No:0)	2.984	0.694-2.836	0.142
Diabetes (Yes: 1; No:0)	1.517	0.060-4.442	0.548
Arterial Hypertension (Yes: 1; No:0)	1.916	0.157-2.380	0.610
Mediterranean Diet (Yes: 1; No:0)	0.599	0.135-2.668	0.501
Physical Exercise (Yes: 1; No:0)	0.717	0.155-3.317	0.670
Anticoagulant/antiaggregant drugs (Yes: 1; No:0)	0.849	0.406-2.148	0.730
GLPI-RA administration (Yes: 1; No:0)	0.781	0.567-0.982	0.082
Statins administration (Yes:1: No:0)	0.646	0.571-0.893	0.061

HDL: High-density lipoprotein; LDL: Low-density lipoprotein; HbA1c: Glycosylated Hemoglobin; SBP: Systolic blood pressure; DBP: Diastolic blood pressure; NFS: NAFLD Fibrosis score; FIB-4: Fibrosis-4; BARD: BMI-AST/Platelet Ratio-Diabetes; LSM: Liver stiffness measurement; BMI: Body Mass Index: GLPI-RA: Glucagon-like peptide-1 receptor-agonists.
