# Supplementary material for: FLAME: Training and Validating a Newly Conceived Model Incorporating Alpha-Glutathione-S-Transferase Serum Levels for Predicting Advanced Hepatic Fibrosis and Acute Cardiovascular Events in Metabolic Dysfunction-Associated Steatotic Liver Disease (MASLD)
Source: Int J Mol Sci. 2025 Jan 17;26(2):761. doi: 10.3390/ijms26020761 (PMC11765617; doi:10.3390/ijms26020761)
Supplement: Supplementary file 1 [file ijms-26-00761-s001.zip › Supplementary Table S3.pdf]

Supplementary Table 3A. Liver-related Events distribution (splitting for HCC) in MASLD patients during 5-year follow-up: detailed reporting (Training Cohort)

**Number of events (Total): 77<br>First LREs: 59<br>HCC occurrence: 18**		**Frequency distribution according to baseline liver fibrosis stage (N. of patients)**		**Chi-square test (F0F2 vs F3F4)**
**Hepatocellular carcinoma occurence**	18	**F0-F2 (n:85)**	8	**p<0.0001**
		**F3-F4 (n:103)**	10	
**Ascites**	37	**F0-F2 (n:85)**	6	**p<0.0001**
		**F3-F4 (n:103)**	31	
**Hepatic encephalopathy**	14	**F0-F2 (n:85)**	2	**p<0.0001**
		**F3-F4 (n:103)**	12	
**Gastroesophageal bleeding**	4	**F0-F2 (n:85)**	0	/
		**F3-F4 (n:103)**	4	
**Jaundice**	4	**F0-F2 (n:85)**	0	/
		**F3-F4 (n:103)**	4	

12 individuals were lost (5 F0-F2 and 7 F3-F3 individuals dead of extrahepatic and extra-cardiovascular events). *HCC: Hepatocellular carcinoma; CSPH: Clinically Significant portal hypertension FUP: follow-up;*Chi-square test. Statistically significant differences (p<0.05) are reported in bold; n.s: not statistically significant.*

Supplementary table 3B. Liver-related Events distribution (splitting for HCC) during 5-year follow-up: detailed reporting (Validation Cohort)

**Number of events (Total): 28**<br>**First LREs: 19**<br>**HCC occurrence: 9**		**Frequency distribution according to baseline liver fibrosis stage (Number of patients)**		**Chi-square test (F0F2 vs F3F4)**
**Hepatocellular carcinoma occurence**	9	**F0-F2 (n:27)**	1	**p<0.0001**
		**F3-F4 (n:33)**	8	
**Ascites**	8	**F0-F2 (n:27)**	1	**p<0.0001**
		**F3-F4 (n:33)**	7	
**Hepatic encephalopathy**	5	**F0-F2 (n:27)**	1	**p<0.0001**
		**F3-F4 (n:33)**	4	
**Gastroesophageal bleeding**	3	**F0-F2 (n:27)**	0	/
		**F3-F4 (n:33)**	3	
**Jaundice**	3	**F0-F2 (n:27)**	0	/
		**F3-F4 (n:33)**	3	

*HCC: Hepatocellular carcinoma. *Chi-square test. Statistically significant differences (p<0.05) are reported in bold; n.s: not statistically significant*
