# Supplementary material for: FLAME: Training and Validating a Newly Conceived Model Incorporating Alpha-Glutathione-S-Transferase Serum Levels for Predicting Advanced Hepatic Fibrosis and Acute Cardiovascular Events in Metabolic Dysfunction-Associated Steatotic Liver Disease (MASLD)
Source: Int J Mol Sci. 2025 Jan 17;26(2):761. doi: 10.3390/ijms26020761 (PMC11765617; doi:10.3390/ijms26020761)
Supplement: Supplementary file 1 [file ijms-26-00761-s001.zip › Supplementary Table S4.pdf]

Supplementary Table S4A. The "ongoing" therapies/medications received by each patient (Training Cohort).

ID	Medications	ID	Medications	ID	Medications	ID	Medications	ID	Medications
**1**	Insulin; (1); (3)	**36**	Diuretics; CCB	**71**	Insulin; (1)	**106**	Statin; Ezetimibe	**141**	Insulin; (3)
**2**	Metformin; (2)	**37**	Diuretics; (2);	**72**	Insulin; (4); (3)	**107**	Diuretics; ACE-i;	**142**	Metformin; (4)
**3**	Metformin; Ezetimibe	**38**	Insulin; (3); (8)	**73**	Insulin; (3)	**108**	Diuretics; (4); (0)	**143**	Metformin; Ezetimibe
**4**	Statin; Ezetimibe	**39**	Metformin; (1); (3)	**74**	Metformin + Vildagliptin	**109**	Diuretics; (6A); (9)	**144**	Diuretics; (9); (2)
**5**	Diuretics; ACE-i;	**40**	Metformin; (0)	**75**	Insulin; (4); (3)	**110**	(10)	**145**	Ezetimibe ;(1)
**6**	CCB; (1); (3); (5)	**41**	(8) + (10)	**76**	Insulin; (1); (3)	**111**	Statin; (9)	**146**	Metformin + Sitagliptin;(0)
**7**	Diuretics; ACE-i;	**42**	Diuretics; CCB	**77**	Insulin; (2)	**112**	(10)	**147**	Insulin; (0)
**8**	ACE-i; (1); (2); (4)	**43**	ACE-i; (0)	**78**	Metformin; (9)	**113**	Diuretics; (3);	**148**	Insulin; (1); (3)
**9**	Diuretics; CCB; (0)	**44**	Statin; Ezetimibe	**79**	Metformin; statin	**114**	Insulin; (1); (3)	**149**	Insulin; (3); (0)
**10**	Diuretics; (1); (3)	**45**	Insulin; Ezetimibe	**80**	Metformin; (2)	**115**	Metformin; (5)	**150**	Statin; Ezetimibe; (5)
**11**	Insulin; (1); (3)	**46**	Metformin; (0)	**81**	Insulin; Diuretics; ACE-i	**116**	Metformin + glimepiride		
**12**	Metformin + Sitagliptin; (2); (5)	**47**	Metformin; (1); (3)	**82**	Insulin; (5)	**117**	Insulin; Ezetimibe; (3)		

**13**	Insulin	**48**	Diuretics; ACE-i;(0)	**83**	Insulin; statin; (1); (3)	**118**	(10)
**14**	ACE-i; (1); (3)	**49**	Insulin; (1); (3); (5)	**84**	Metformin; (2); (0)	**119**	Statin; Ezetimibe; (2)
**15**	Diuretics; (2); (0)	**50**	Metformin; Ezetimibe	**85**	Metformin + Glimepiride	**120**	Diuretics; (6A); (6B)
**16**	Diuretics; CCB	**51**	Metformin; (9)	**86**	Insulin; (0)	**121**	Statin; (1); (3)
**17**	Insulin; Diuretics; ACE-i	**52**	Insulin; Diuretics; ACE-i	**87**	Insulin; (3); (0)	**122**	(1); (3); (6A)
**18**	Diuretics; (7A); (7B)	**53**	Diuretics; (0)	**88**	Insulin; (5)	**123**	Diuretics; (5)
**19**	Insulin; (1); (4)	**54**	Diuretics; (8)	**89**	Metformin + Sitagliptin	**124**	CCB; Diuretics
**20**	Metformin + Sitagliptin	**55**	Insulin; statin; (5); (0)	**90**	Insulin; (2); (0)	**125**	Insulin
**21**	Insulin; (8)	**56**	Insulin; (1); (3)	**91**	Insulin; Ezetimibe; (9)	**126**	Insulin; (9)
**22**	Statin; Ezetimibe	**57**	Insulin; (3); (1)	**92**	(4); (5)	**127**	Insulin; (8); (2)
**23**	Statin; (1); (3)	**58**	Insulin; (1); (3)	**93**	Diuretics; (3)	**128**	Metformin; (9)
**24**	Statin; Fibrate	**59**	Insulin; (1); (3)	**94**	Insulin; statin; (9)	**129**	Insulin; (5)
**25**	Insulin; (4); (8)	**60**	Insulin; (8)	**95**	Metformin + Sitagliptin	**130**	Insulin; (9); (0)
**26**	Insulin; (2)	**61**	Metformin; Ezetimibe	**96**	Insulin; (9); (4)	**131**	Diuretics; (3)
**27**	Insulin; Ezetimibe	**62**	Statin; Ezetimibe	**97**	CCB; (3); (0)	**132**	CCB; (3); (1)

**28**	Metformin + Vildagliptin	**63**	Insulin	**98**	Diuretics; CCB	**133**	Statin; Ezetimibe
**29**	Insulin; (2)	**64**	Metformin + Vildagliptin	**99**	Statin; Ezetimibe	**134**	Diuretics; CCB
**30**	Insulin; (4); (0)	**65**	Insulin; (1); (3)	**100**	Metformin + Glimepiride	**135**	Insulin; (6A);
**31**	Insulin; Ezetimibe	**66**	Diuretics; (4)	**101**	Diuretics; (0)	**136**	Insulin; (0)
**32**	Metformin + Sitagliptin	**67**	Diuretics; (8)	**102**	Insulin; statin; Fibrate	**137**	Insulin; Ezetimibe
**33**	Insulin; (1); (3)	**68**	CCB; (6A)	**103**	CCB; (9); (3)	**138**	Metformin; statin
**34**	Metformin; (8)	**69**	Statin; Ezetimibe; Fibrate	**104**	CCB; (1); (3)	**139**	Insulin; (6B)
**35**	Insulin; (3)	**70**	Diuretics; CCB	**105**	Diuretics;	**140**	Insulin; statin; (0)

Supplementary Table S4B. The “ongoing” therapies/medications received by each patient (Validation Cohort).

ID	Medications	ID	Medications	ID	Medications
**1**	Metformin + Glimepiride	**13**	Diuretics; CCB	**25**	Insulin; (1)
**2**	Diuretics; (0)	**14**	Diuretics; (2);	**26**	Insulin; (3)
**3**	Metformin; Ezetimibe	**15**	Insulin; (8)	**27**	Insulin; (3)
**4**	Statin; Ezetimibe	**16**	Metformin; (1); (3)	**28**	Metformin
**5**	Diuretics; ACE-i;	**17**	(0)	**29**	Insulin; (3)
**6**	Metformin + Vildagliptin	**18**	(8) + (10)	**30**	Insulin; (1); (3)
**7**	Insulin; (2)	**19**	Diuretics; CCB	**31**	Insulin; (2)
**8**	Insulin; (4); (0)	**20**	ACE-i; (0)	**32**	Metformin; (9)
**9**	Metformin + Vildagliptin	**21**	Metformin; (0)	**33**	Metformin; statin
**10**	Diuretics; (1); (3)	**22**	Insulin; Ezetimibe	**34**	Metformin; (2)
**11**	Insulin; (1); (3)	**23**	Metformin; (0)	**35**	Insulin; Diuretics; ACE-i
**12**	Metformin + Sitagliptin; (2); (5)	**24**	Statin; Ezetimibe.	**36**	Insulin; (5)

LEGEND

Medications without brackets were already administered at the baseline; medications in brackets were added during the follow-up.

ID patients with white backgrounds were at the baseline drug-free individuals. 50 individuals in the training cohort were drug-free (at baseline and during follow-up) and thus medications were not reported. 24 individuals in the validation cohort were drug-free (at baseline and during follow-up) and thus medications were not reported.

Abbreviations: *CCB (calcium channel blockers); ACE-i: Angiotensin Converting Enzyme inhibitors*.

*** "Others" medications synthetically and schematically included:**
(0) Glucagon-like peptide-1 receptor agonist (2) Antibiotics (excluding Rifaximin)
(3) Statins
(4) Antiaggregant and/or anticoagulant
(5) Non-steroidal anti-inflammatory drugs (NSAIDs)
(6A) Systemic steroids
(6B) Local/Topic steroids
(7A) Selective Serotonin Reuptake Inhibitors
(7B) Benzodiazepine (BDZ)
(8) Proton Pump Inhibitors (PPI)
(9) Laxatives (Excluding lactulose)
(10) Others/Not specifically declared or reported by the relative ID patient
