# Supplementary material for: FLAME: Training and Validating a Newly Conceived Model Incorporating Alpha-Glutathione-S-Transferase Serum Levels for Predicting Advanced Hepatic Fibrosis and Acute Cardiovascular Events in Metabolic Dysfunction-Associated Steatotic Liver Disease (MASLD)
Source: Int J Mol Sci. 2025 Jan 17;26(2):761. doi: 10.3390/ijms26020761 (PMC11765617; doi:10.3390/ijms26020761)
Supplement: Supplementary file 1 [file ijms-26-00761-s001.zip › Supplementary Table S5.pdf]

Supplementary Table S5. Medical validated questionnaire and relative items assessing physical exercise practicing in MASLD enrolled patients.

**Questions/Items**	**Answer**	
Are you doing or have you ever done (in the last 2 years) sport in a continuative and regular way?	YES	NO
Have you changed your daily physical activity in the last 6 months?	NO	YES
If yes, has it enhanced or worsened?	Enhanced	Worsened
How many hours per week do you usually spend for physical exercise?	More than 150 minutes/week	Less than 150 minutes/week

Each patient was considered on **active** physical exercise if he/she has done sports in the last 2 years, this practice has not worsened in the last 6 months by spending at least 150 minutes per week in physical activity.
