# Supplementary material for: FLAME: Training and Validating a Newly Conceived Model Incorporating Alpha-Glutathione-S-Transferase Serum Levels for Predicting Advanced Hepatic Fibrosis and Acute Cardiovascular Events in Metabolic Dysfunction-Associated Steatotic Liver Disease (MASLD)
Source: Int J Mol Sci. 2025 Jan 17;26(2):761. doi: 10.3390/ijms26020761 (PMC11765617; doi:10.3390/ijms26020761)
Supplement: Supplementary file 1 [file ijms-26-00761-s001.zip › Supplementary Table S6.pdf]

**Supplementary Table S6.** The Mediterranean diet score: items and scoring system.

| ITEM: Number | Question | Answer | |
|---|---|---|---|
| 1 | **How often do you eat vegetables?** | <4 servings/day | ≥4 servings/day |
| 2 | **How often do you eat fruit?** | <3 servings/day | ≥3 servings/day |
| 3 | **How often do you eat legumes?** | 0 serving/week | ≥ 1 serving/week |
| 4 | **How often do you take Nuts and Seeds?** | 0 serving/week | ≥ 1 serving/week |
| 5 | **How often do you take whole grains?** | 0 serving/day | ≥ 1 serving/day |
| 6 | **How often do you eat fish?** | <3 servings/week | ≥4 servings/week |
| 7 | **What type of fat do you eat the most?** | Mostly saturated (butter, eggs, confectionery, baked goods) | Mainly monounsaturated and polyunsaturated (extra virgin olive oil, seeds, nuts, oily fish) |
| 8 | **How much alcohol do you drink per day** | No consumption | ≥1 glass/day |
| 9 | **How often do you eat red and processed meats?** | <3 servings/day | ≥3 servings/day |

**Items scoring and interpretation of the results**: 1) "How often do you eat vegetables?" (< 4 servings per day: 0 points; ≥ 4 servings per day: 1 point); 2) "How often do you eat fruit?" (< 3 servings per day: 0 points; ≥ 3 servings per day: 1 point); 3) "How often do you eat legumes?" (0 servings per week: 0 points; ≥ 1 serving per week: 1 point); 4) "How often do you take Nuts and Seeds?" (0 servings per week: 0 points; ≥ 1 serving per week: 1 point); 5) "How often do you take

whole grains?” (0 servings per day: 0 points; ≥ 1 serving per day: 1 point); 6) “How often do you eat fish?” (< 3 servings per week: 0 points; ≥ 4 servings per week: 1 point); 7) “What type of fat do you eat the most?” [Mostly saturated (butter, eggs, confectionery, baked goods)]: 0 points; [Mainly monounsaturated and polyunsaturated (extra virgin olive oil, seeds, nuts, oily fish)]: 1 point; 8) “How much alcohol do you drink per day?” (No consumption: 0 points; More than one glass a day: 1 point); 9) “How often do you eat red and processed meats?” (≥ 3 servings per day: 0 points; < 3 servings per day: 1 point). **The patients were considered adherent to the Mediterranean diet regimen in case of a total score > 8.**
