# Supplementary figures and images for: FLAME: Training and Validating a Newly Conceived Model Incorporating Alpha-Glutathione-S-Transferase Serum Levels for Predicting Advanced Hepatic Fibrosis and Acute Cardiovascular Events in Metabolic Dysfunction-Associated Steatotic Liver Disease (MASLD)
Source: Int J Mol Sci. 2025 Jan 17;26(2):761. doi: 10.3390/ijms26020761 (PMC11765617; doi:10.3390/ijms26020761)

**A**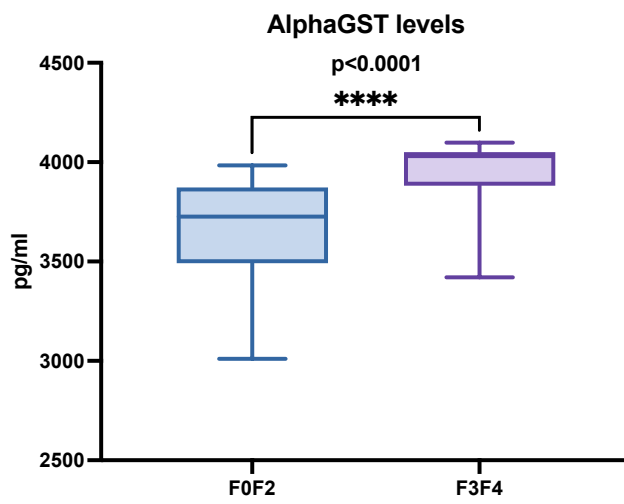**B**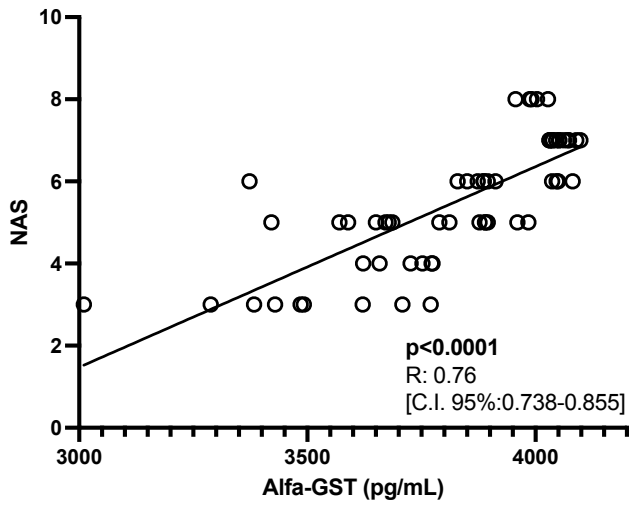**C**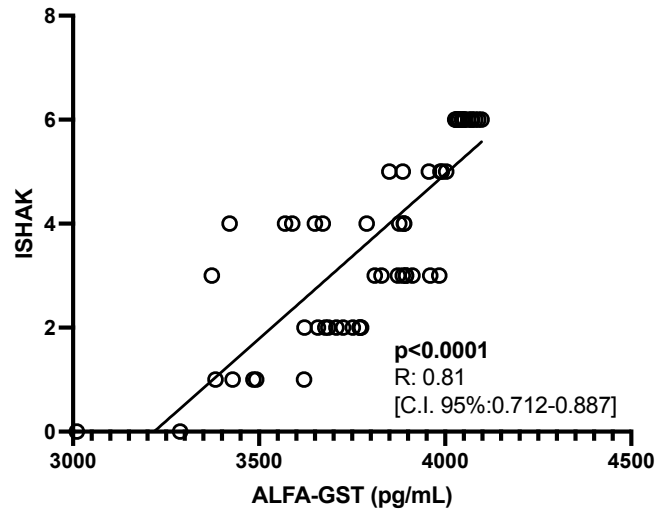**D**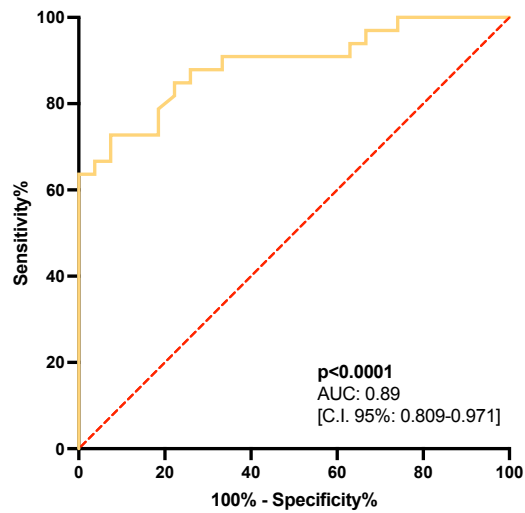

Supplement: Supplementary file 1 [file ijms-26-00761-s001.zip › Supplementary Figure S2.pdf]

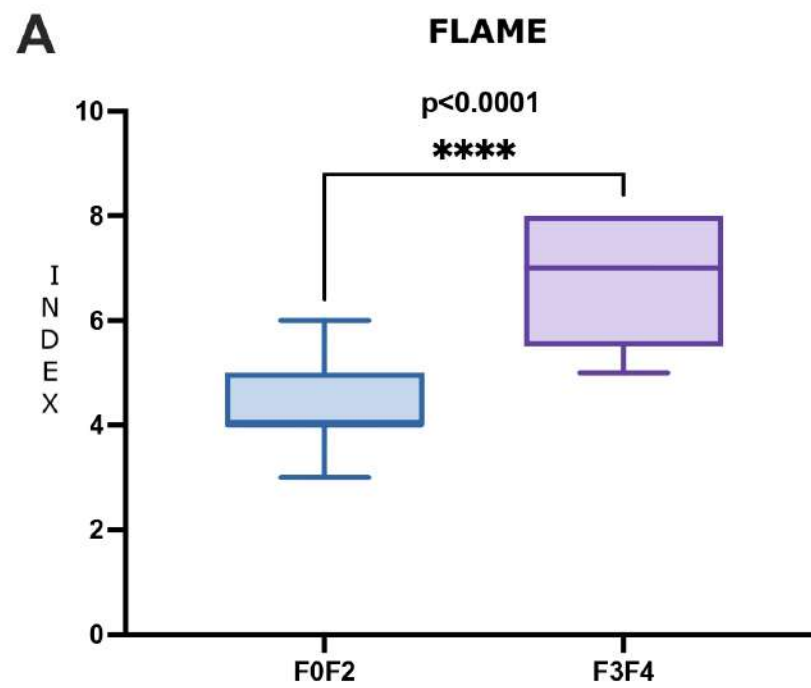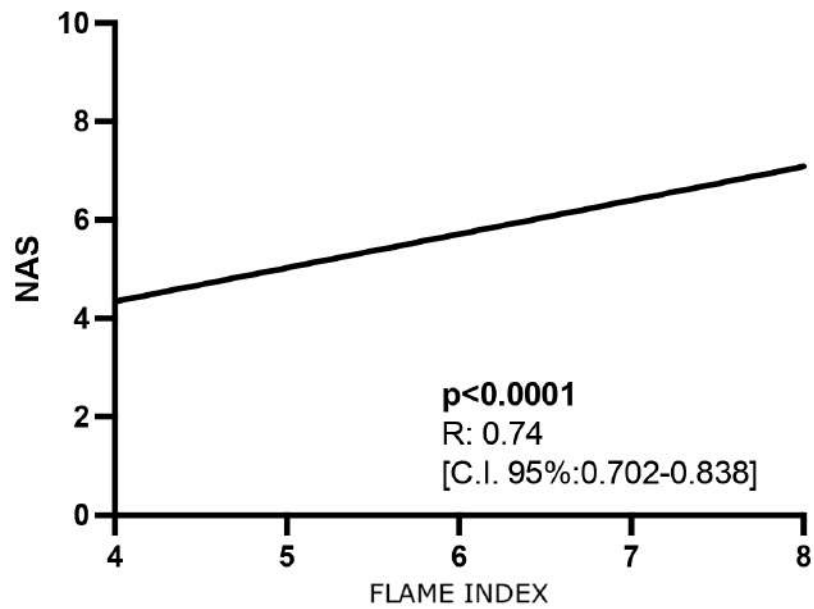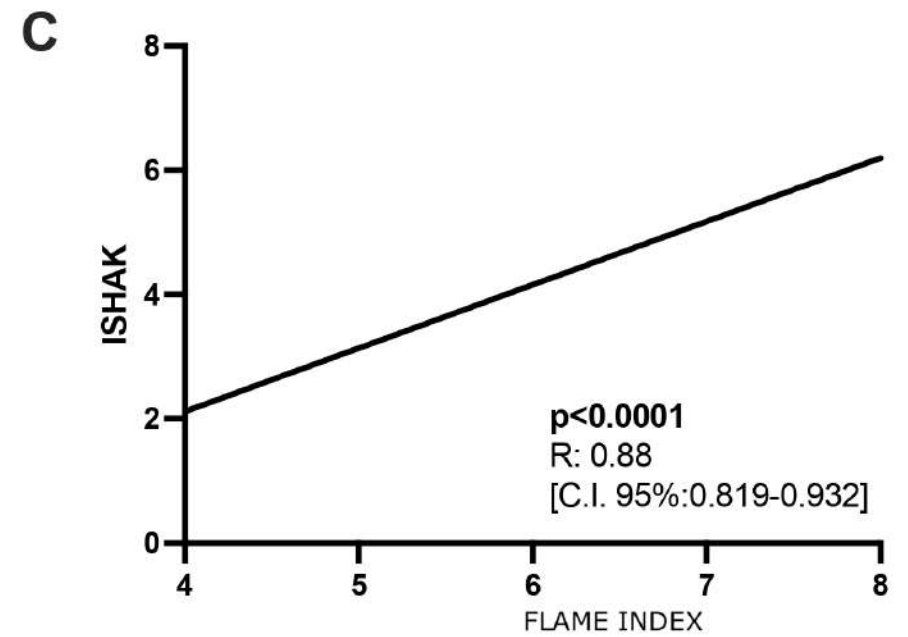

Supplement: Supplementary file 1 [file ijms-26-00761-s001.zip › Supplementary Figure S3.pdf]
